# A stable cathode-solid electrolyte composite for high-voltage, long-cycle-life solid-state sodium-ion batteries

**DOI:** 10.1038/s41467-021-21488-7

**Published:** 2021-02-23

**Authors:** Erik A. Wu, Swastika Banerjee, Hanmei Tang, Peter M. Richardson, Jean-Marie Doux, Ji Qi, Zhuoying Zhu, Antonin Grenier, Yixuan Li, Enyue Zhao, Grayson Deysher, Elias Sebti, Han Nguyen, Ryan Stephens, Guy Verbist, Karena W. Chapman, Raphaële J. Clément, Abhik Banerjee, Ying Shirley Meng, Shyue Ping Ong

**Affiliations:** 1 Department of NanoEngineering, University of California San Diego, La Jolla, San Diego, CA USA; 2 Materials Department, University of California Santa Barbara, Santa Barbara, CA USA; 3 Department of Chemistry, Stony Brook University, Stony Brook, New York, NY USA; 4 Department of Materials Science and Engineering, University of California San Diego, La Jolla, San Diego, CA USA; 5 Department of Chemistry & Biochemistry, University of California San Diego, La Jolla, San Diego, CA USA; 6 Shell International Exploration & Production Inc., Houston, TX USA; 7 Shell Global Solutions International BV, The Hague, Netherlands; 8 Research Institute for Sustainable Energy (RISE), TCG Centres for Research and Education in Science and Technology (TCG CREST), Sector V, Salt Lake, Kolkata, India; 9 Sustainable Power & Energy Center (SPEC), University of California San Diego, La Jolla, San Diego, CA USA

**Keywords:** Batteries, Engineering, Batteries

## Abstract

Rechargeable solid-state sodium-ion batteries (SSSBs) hold great promise for safer and more energy-dense energy storage. However, the poor electrochemical stability between current sulfide-based solid electrolytes and high-voltage oxide cathodes has limited their long-term cycling performance and practicality. Here, we report the discovery of the ion conductor Na_3-x_Y_1-x_Zr_x_Cl_6_ (NYZC) that is both electrochemically stable (up to 3.8 V vs. Na/Na^+^) and chemically compatible with oxide cathodes. Its high ionic conductivity of 6.6 × 10^−5^ S cm^−1^ at ambient temperature, several orders of magnitude higher than oxide coatings, is attributed to abundant Na vacancies and cooperative MCl_6_ rotation, resulting in an extremely low interfacial impedance. A SSSB comprising a NaCrO_2_ + NYZC composite cathode, Na_3_PS_4_ electrolyte, and Na-Sn anode exhibits an exceptional first-cycle Coulombic efficiency of 97.1% at room temperature and can cycle over 1000 cycles with 89.3% capacity retention at 40 °C. These findings highlight the immense potential of halides for SSSB applications.

## Abstract (web summary)

Rechargeable solid-state sodium-ion batteries hold great promise for safer and more energy-dense energy storage. Here, the authors show a new sodium-based halide, Na_3-x_Y_1-x_Zr_x_Cl_6_, for sodium-all-solid-state batteries with enhanced ionic conductivity and long-term cycling stability.

## Introduction

A solid-state architecture for rechargeable sodium-ion batteries has garnered substantial research interest in recent years^1–5^. By replacing flammable organic liquid electrolytes with solid electrolytes (SEs), solid-state sodium-ion batteries (SSSB) promise not only higher safety, but also potentially enable higher voltage cathodes, metal anodes, and stacking architectures to achieve higher energy densities. In addition, the higher abundance of sodium relative to lithium makes sodium-ion batteries a more cost-effective alternative, especially for large-scale grid storage applications where low operating costs are more strongly emphasized than a high energy density^6^. However, an ideal SE has to meet a stringent set of requirements, namely high ionic conductivity, low electronic conductivity, and electrochemical, chemical, and mechanical compatibility with electrodes. While major breakthroughs have been made in achieving liquid-like ionic conductivity values in sulfide SEs, their poor electrochemical and chemical interfacial stability against common electrodes remains a critical bottleneck for practical applications.

Recently, two lithium halide superionic conductors, Li_3_YCl_6_ and Li_3_YBr_6_, have been reported as promising SEs for solid-state lithium-ion batteries^7^. Exhibiting reasonable Li^+^ conductivities in the range of 0.5-0.7 mS cm^−1^, the most interesting feature of these halide SEs is their electrochemical and chemical stability, demonstrated via compatibility with the 4 V LiCoO_2_ cathode^7^. As a result, more studies have since emerged on halide SEs (Li_3_InCl_6_ and Li_*x*_ScCl_3+*x*_) that also exhibit high Li^+^ diffusivity, compatibility with LiCoO_2_, and facile processability^8–10^. In addition to these, there has also been a report on aliovalent substitution with Zr, yielding the halide Li_3−*x*_M_1−*x*_Zr_*x*_Cl_6_ (M = Er, Y), where the introduction of vacancies led to an increase in the ionic conductivity up to the order of 10^−3^ S/cm at room temperature^11^. Interestingly, unlike the fast Li-ion conducting sulfides or oxides, fast Li-ion conduction in these halide frameworks do not require a bcc anion sublattice, allowing a much wider selection of compositions when designing halide SE chemistries^10^. It is important to note that to a first approximation, the oxidative electrochemical stability of SEs are determined by anion chemistry^10,12,13^, and for halides, it generally follows the trend F > Cl>Br>I^10,13^.

In contrast to the Li halides mentioned, the Na analogs Na_3_YCl_6_ and Na_3_YBr_6_ have been relatively less studied; previous studies have reported experimental ionic conductivities on the order of 10^−4^–10^−6^ S/cm at 500 K. These materials are therefore expected to have much lower room-temperature ionic conductivities than their lithium counterparts and thus impractical for SE applications^14^. Here, we report the data-driven development of Na_3-*x*_Y_1-*x*_Zr_*x*_Cl_6_ (NYZC*x*) as a new class of sodium SEs exhibiting high ionic conductivities as well as excellent electrochemical and chemical stability up to 3.8 V vs Na/Na^+^. Using density functional theory (DFT) calculations, it was predicted that aliovalent doping of Y^3+^ with Zr^4+^ would improve the Na^+^ conductivity of Na_3_YCl_6_ by three orders of magnitude, while retaining a wide electrochemical window and good chemical stability. A SSSB comprising a NaCrO_2_:NYZC0.75:vapor grown carbon fibers (VGCF) composite cathode with Na_3_PS_4_ (NPS) as the SE and a Na-Sn (2:1) anode exhibited an extremely high first cycle Coulombic efficiency (CE) of 97.6% at room temperature. Even when cycled at 40 °C and a rate of 1 C, the SSSB displayed stable electrochemical performance over 1000 cycles with 89.3% capacity retention.

## Results and discussion

### Electrochemically stable and conductive Na_3-*x*_Y_1-*x*_Zr_*x*_Cl_6_

**Fig. 1:**
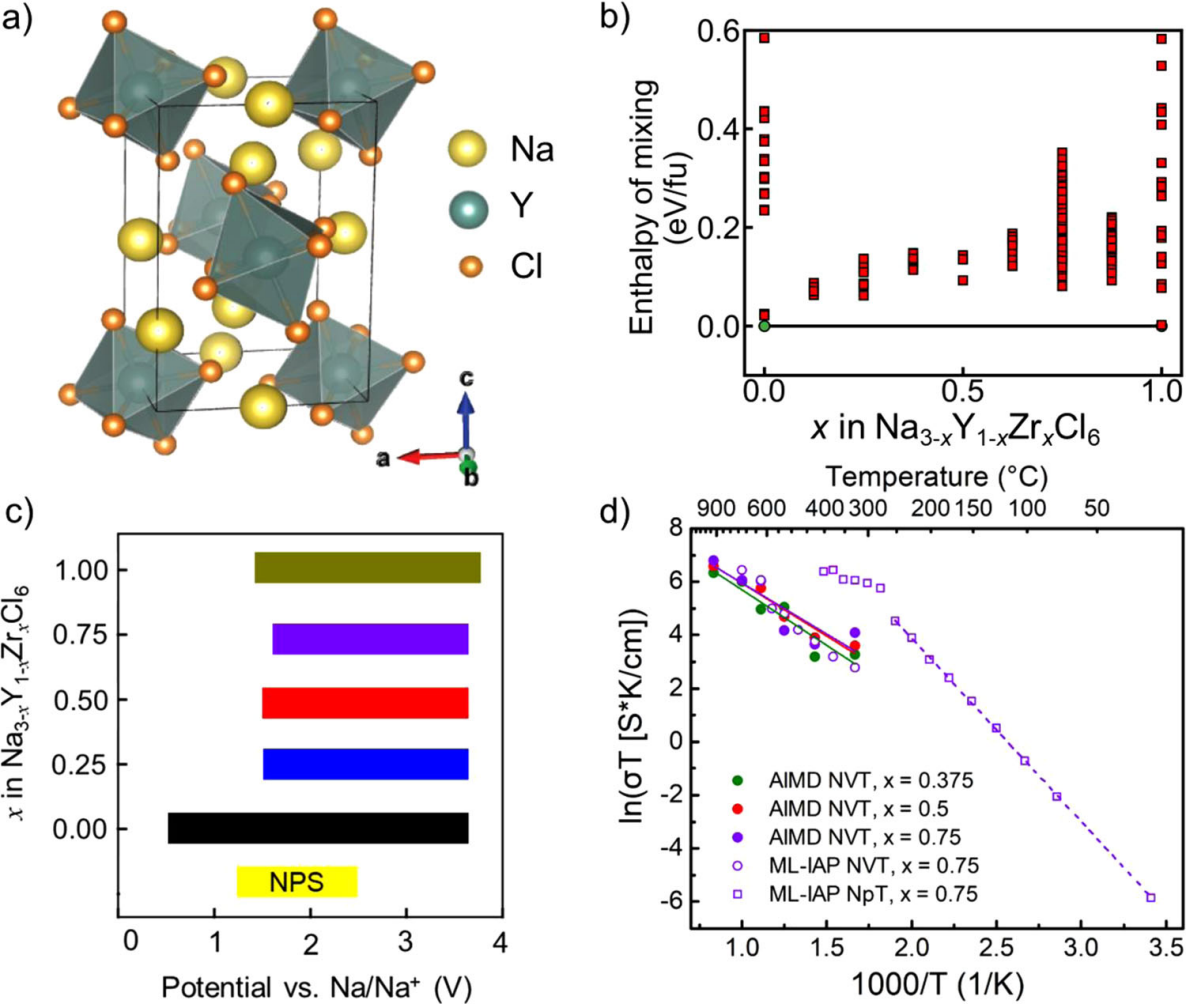
Effect of Zr dopants on properties of Na_3_YCl_6_. **a** Crystal structure of Na_3_YCl_6_. **b** Stability of Na_3-*x*_Y_1-*x*_Zr_*x*_Cl_6_ after incorporating Zr^4+^ into Na_3_YCl_6_. Each square marker indicates a symmetrically distinct ordering of Na and Y/Zr. **c** Electrochemical stability window of Na_3-*x*_Y_1-*x*_Zr_*x*_Cl_6_ (0 ≤ *x* ≤ 1), with the window of Na_3_PS_4_ (NPS) shown as a reference. **d** Arrhenius plot for Na_3-*x*_Y_1-*x*_Zr_*x*_Cl_6_ from AIMD simulations (at *x* = 0.375, 0.5, and 0.75; solid lines and markers) and ML-IAP MD simulations (at *x* = 0.75; dashed lines and open markers). AIMD simulations were carried out at *T* = 600–1000 K at 100 K intervals, using a supercell of 150 atoms for up to 200 ps, while the ML-IAP MD simulations were carried out at *T* = 350 K–650 K using a supercell of 592 atoms for up to 10 ns.

Unlike its lithium counterpart_,_ Na_3_YCl_6_ (NYC) (Fig. 1a, space group: P2_1_/n) does not exhibit partial occupancy in the 2d and 4e Na sites, which may explain its lower ionic conductivity. A series of ions (Ti^4+^, Zr^4+^, Hf^4+^, and Ta^5+^) were evaluated as potential aliovalent dopants for Y^3+^ to increase the concentration of defects and thus the ionic conductivity of Na_3-(*z*-3)*x*_Y^3+^_1-*x*_M^z+^_*x*_Cl_6_^2,4,15–17^. The effect of ionic substitution on the phase stability of NYC is shown in Supplementary Figure 1. Zr^4+^ is predicted to exhibit a low dopant formation energy and is also low-cost, due to the abundance of Zr. Furthermore, the enthalpies of mixing of the NYC-Na_2_ZrCl_6_ (NZC) pseudo-binary system are low, as shown in Fig. 1b.

The electrochemical window of Na_3-*x*_Y_1-*x*_Zr_*x*_Cl_6_ (NYZC*x*) was investigated using the grand potential phase diagram approach^18,19^. Consistent with previous studies on the Li analogs, NYZC*x* SEs exhibit wide electrochemical windows, with a particularly high oxidation limit of ~3.8 V vs Na/Na^+^ (Fig. 1c). This high oxidation limit is maintained regardless of Zr content. However, the reduction limit narrows (from 0.6 V for NYC to 1.5 V for NYZC*x*), due to the higher thermodynamic reduction potential of Zr^4+^ compared to Y^3+^. The oxidation limit of 3.8 V for NYZC*x* indicates that it could be compatible with the NaCrO_2_ cathode, which has an operating voltage window of 2–3.6 V vs Na/Na^+^^20^. In contrast, sulfide SEs, such as Na_3_PS_4_, have oxidation limits of only ~2.5 V vs Na/Na^+^^21^. In addition, the reaction energies of NYZC0.75 with NaCrO_2_ and with metallic Na were found to be less negative compared to NPS (Supplementary Table 2).

The crystalline form of the end members NYC and NZC exhibit a closed-pack arrangement of [YCl_6_]^3−^ and [ZrCl_6_]^2−^ polyanions, respectively. With increasing *x* in NYZC*x*, the unit cell volume increases, which results in a widening of the Na^+^ diffusion channels, as shown in Supplementary Figure 2. NVT ab initio molecular dynamics (AIMD) simulations were carried out at 600−1000 K for NYZC*x* for *x* = 0, 0.375, 0.5, and 0.75. For NYC, AIMD simulations indicate no diffusion of Na^+^ ions even at elevated temperatures (Supplementary Table 1), consistent with its poor ionic conductivity. With Zr^4+^ doping, Na^+^ diffusivity increases substantially (Fig. 1d).

Due to the high cost of ab initio methods, NVT AIMD simulations (constrained to the pre-equilibrated volume) were limited to small supercells and temperatures above 500 K to ensure sufficient diffusion statistics. To probe the diffusivity at lower temperatures, a highly accurate ML-IAP based on the moment tensor potential formalism was developed using snapshots extracted from the AIMD trajectories as well as ground state and strained structures of NYC, NZC and the highest conductivity NYZC0.75 (see Methods section for details)^22–25^. To our knowledge, this is the first work that demonstrates the use of AIMD simulations to fit a ML-IAP. As shown in Fig. 1d, NVT MD simulations of NYZC0.75 carried out using this ML-IAP reproduces the AIMD diffusivities to good accuracy at 600–1000 K. The validated ML-IAP was then applied for NpT MD simulations of NYZC0.75 using a much larger cell (592 atoms) over much longer time scales (up to 10 ns) and under constant atmospheric pressure at 350 to 650 K. Interestingly, a non-Arrhenius behavior is observed; there is a transition between two linear regimes at around 500–550 K. Similar step changes in diffusion characteristics have been previously observed experimentally in NYC and other superionic conductors^14,26^. The activation barrier for diffusion in the low temperature regime (<500 K) is predicted to be 594 meV, and the room temperature Na^+^ conductivity is predicted to be 1.4 × 10^−5^ S cm^−1^, which is two orders of magnitude higher than that of NYC.

NYZC*x* compounds were synthesized using stoichiometric amounts of NaCl, YCl_3_, and ZrCl_4_ (see Methods). The parent compound NYC was first synthesized (detailed in Supplementary Note 1) and its XRD pattern and corresponding Rietveld Refinement results are shown in Supplementary Fig. 3a and Supplementary Table 3. These results are consistent with previous reports^27^. The room temperature ionic conductivity of NYC was determined to be 9.5 × 10^−8^ S cm^−1^ via electrochemical impedance spectroscopy (EIS) measurements (Supplementary Fig. 3b).

**Fig. 2:**
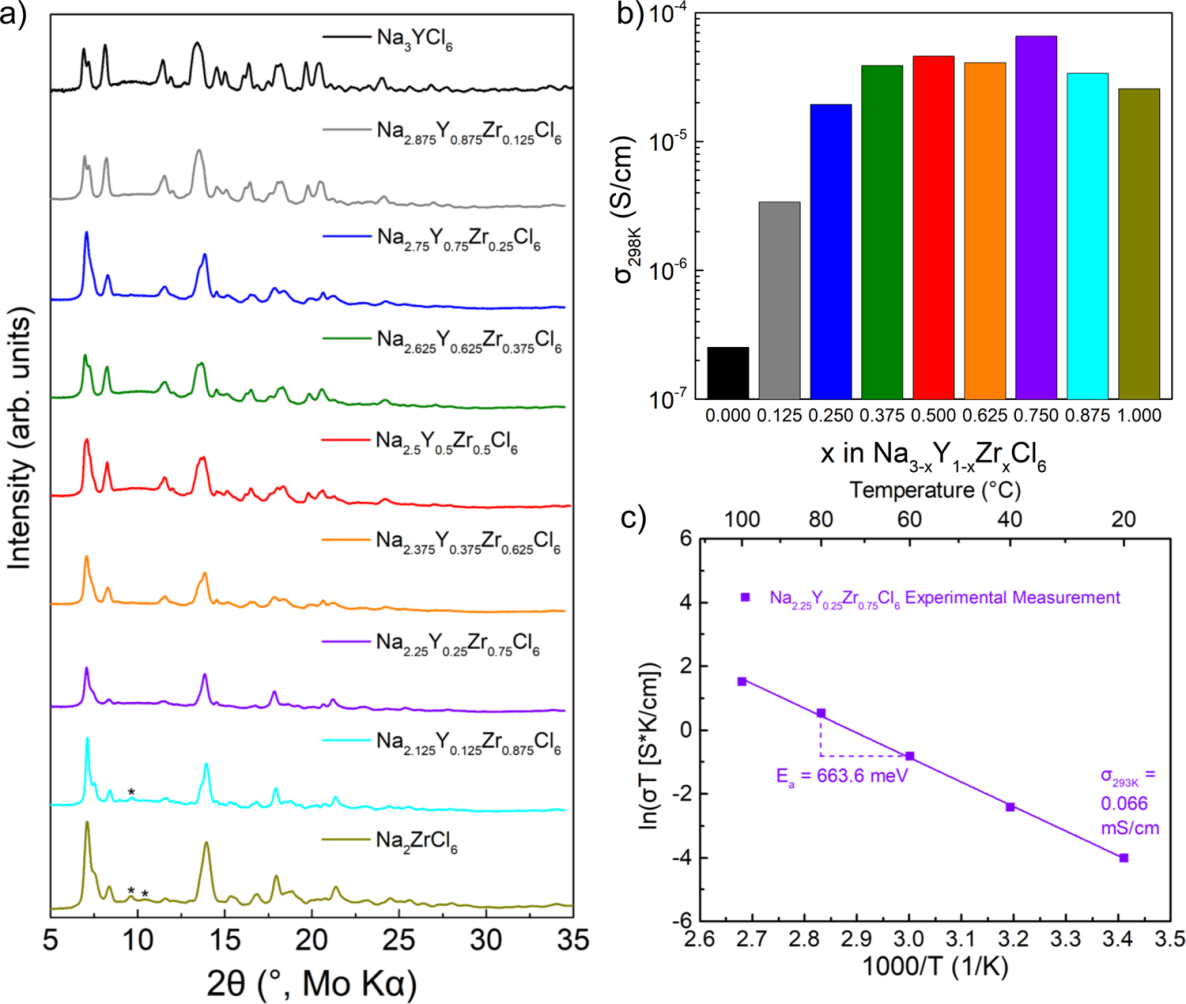
Experimental Characterization of Na_3-*x*_Y_1-*x*_Zr_*x*_Cl_6_. **a** XRD of the Na_3-x_Y_1-x_Zr_x_Cl_6_ compositions, obtained in *x* = 0.125 increments. Asterisks indicate the presence of new peaks. **b** The corresponding room temperature conductivity values. **c** Arrhenius plot of Na_2.25_Y_0.25_Zr_0.75_Cl_6_ from experimental measurements. The activation energy (low-temperature regime) and room temperature conductivity values are consistent with the MTP results.

With Zr doping, the P2_1_/n space group of the parent compound NYC is largely retained (Fig. 2a) up to *x* = 0.875, suggesting a solid solution in this compositional range. For *x* ≥ 0.875, additional peaks emerge in the XRD patterns at 2θ = 9.6° and 10.5°, indicating the presence of a second, different phase. This particular phase was determined to be crystalline NZC; using DFT calculations (Supplementary Note 2 and Supplementary Fig. 4a), the lowest energy NZC structure was found to be isostructural with Na_2_TiF_6_ (hexagonal space group P -3 m 1, structures shown in Supplementary Fig. 4b)^28^. This structure is consistent with experimental synchrotron XRD (λ = 0.1668 Å) and Rietveld refinement results of NZC measured after heat treatment (Supplementary Fig. 4c and Supplementary Table 4). Furthermore, X-ray photoelectron spectroscopy (XPS) measurements (Supplementary Fig. 5) indicate the presence of both Zr-Cl and Y-Cl bonds (as seen in the Cl *2p* region) and thus structural units in the as-prepared NYZC0.5.

Figure 2b shows the extracted conductivity values (from the corresponding Nyquist plots in Supplementary Fig. 6a), over the entire NYZC*x* compositional range at *x* = 0.125 increments. The ionic conductivity for 0.375 ≤ *x* < 1 is in the range of 2.6−6.6 × 10^−5^ S cm^−1^, with NYZC0.75 exhibiting the highest conductivity of 6.6 × 10^−5^ S cm^−1^ (the equivalent circuit fitting is shown in Supplementary Fig. 6b). A drop in conductivity was observed for *x* ≥ 0.875 compositions, attributed to the formation of a small amount of crystalline NZC phase with a much lower room temperature conductivity of 1.4 × 10^−7^ S/cm (Supplementary Fig. 4d). In addition, crystalline NZC has a relative density of ~79%, which could also contribute to its lower conductivity. In contrast, the relative density of NYZC0.75 is 90%, and a cross-sectional scanning electron microscope (SEM) image of the NYZC0.75 pellet indicates a dense particle morphology (Supplementary Fig. 6b inset). Since NYZC0.75 exhibited the highest conductivity among all compositions explored herein, the activation energy was measured (Fig. 2c) and found to be 663.6 meV, in good agreement with the ML-IAP NpT simulations for the low temperature regime. In addition, the electronic conductivity of NYZC0.75 was determined to be 8.89 × 10^−9^ S cm^−1^ via DC polarization (Supplementary Fig. 7), i.e., NYZC0.75 is an ionic conductor and an electronic insulator.

It is important to note that the experimental value of the room temperature conductivity for NYZC0.75 (6.6 × 10^−5^ S cm^−1^) is somewhat higher than the ML-IAP value (1.4 × 10^−5^ S cm^−1^). While crystalline NYZC0.75 was modeled, the ball milling procedure used in our experiments can introduce disorder and amorphize the sample, and it was previously shown that the relatively less crystalline Li_3_YCl_6_ was demonstrated to have a significantly higher conductivity than its highly-crystalline counterpart^7^. Hence, to further determine the impact of crystallinity on Na^+^ diffusion, the ionic conductivity before and after ball milling was investigated for NYC and for NYZC0.75, and results are shown in Supplementary Fig. 8 and detailed in Supplementary Note 3. The local Na environments and structural disorder were also probed via ^23^Na solid-state nuclear magnetic resonance (NMR).

### Local Na environments and disorder

**Fig. 3:**
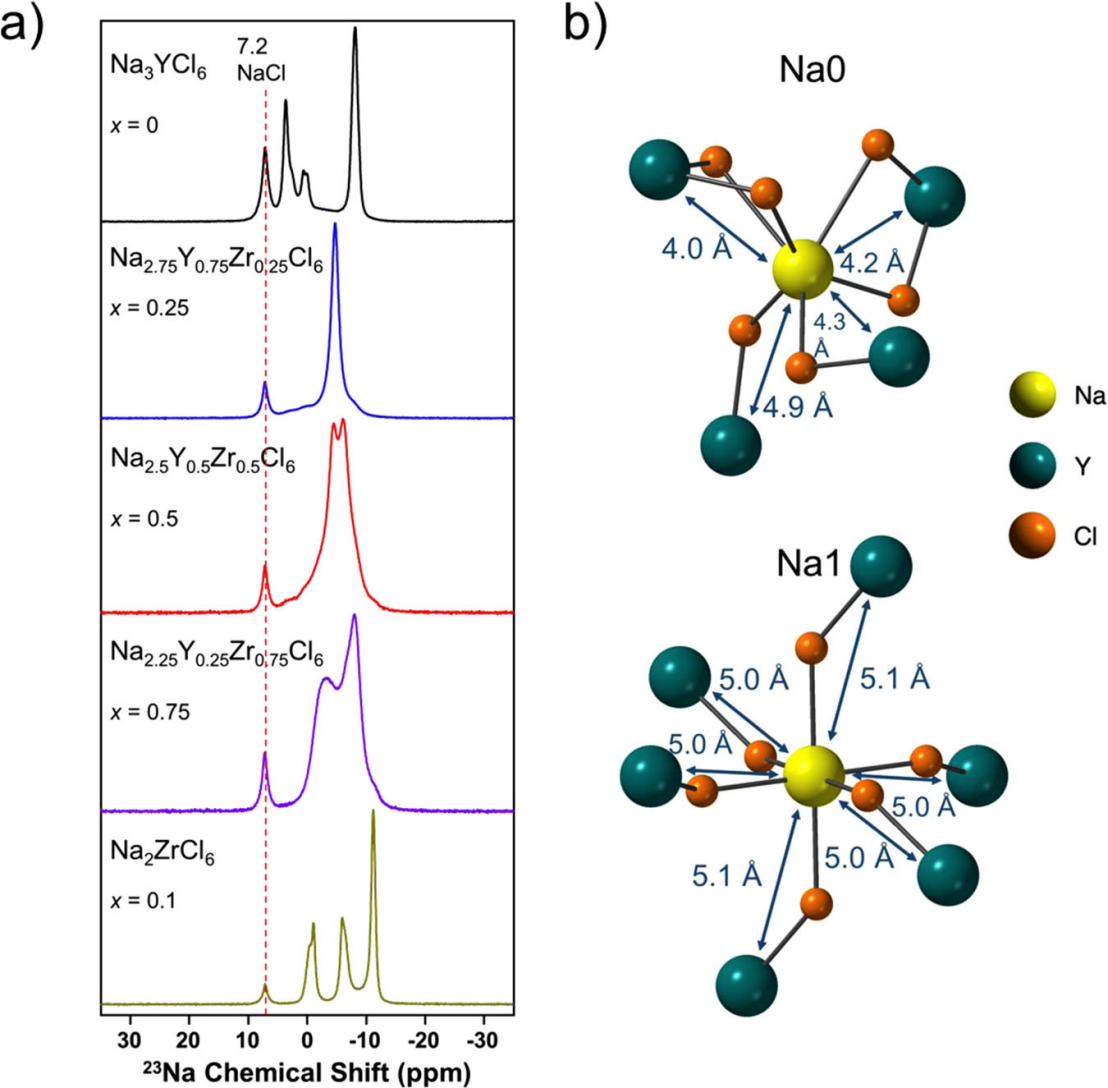
^23^Na single-pulse solid-state NMR spectra. **a** Spectra collected on Na_3-*x*_Y_1-*x*_Zr_*x*_Cl_6_ (*x* = 0, 0.25, 0.5, 0.75 and 1); the data were acquired at 18.8 T and at a MAS rate of 12 kHz and at a set temperature of 298 K. **b** Schematic of the Na0 and Na1 local environments in Na_3_YCl_6_.

^23^Na magic angle spinning (MAS) solid-state NMR spectra obtained on NYZC*x* (*x* = 0, 0.25, 0.5, 0.75, 1) are presented in Fig. 3a. The signal at about 7.2 ppm is attributed to NaCl(s) present as either an impurity or residual precursor phase in all NYZC*x* samples^29^. The NaCl content obtained from ^23^Na NMR signal integration was found to be 16.0 %, 10.0 %, 5.9 %, 6.2 % and 4.0 % for *x* = 0, 0.25, 0.5, 0.75 and 1.0, respectively. The decrease in NaCl impurities with increasing Zr content is likely due to the concomitant decrease in Na content in NYZC*x* relative to NYC, which renders the formation of NaCl less favorable.

While two crystallographically-distinct Na environments are expected in Na_3_YCl_6_ (as shown in Fig. 3b), the spectrum obtained on this compound (top spectrum in Fig. 3) exhibits at least five distinct resonances besides the NaCl impurity signal at about 7.2 ppm, which suggests the presence of local structural disorder and/or non-stoichiometry. We have excluded the possibility of additional impurity phases in the sample, due to the lack of candidate Na-containing impurity phases with ^23^Na resonant frequencies and signal line-shapes matching those observed in the Na_3_YCl_6_ spectrum. The presence of well-defined peaks suggests the presence of a range of Na environments with varying numbers of Cl and Y (Na) nuclei in their first and second coordination shells, respectively, as in a non-stoichiometric material or a material with some disorder on the cation lattice. We note that a non-stoichiometric solid electrolyte phase will necessarily result from the presence of a separate NaCl component, unless some YCl_3_ impurity is also present in the sample. The latter phase is not observed with XRD, but ^89^Y NMR data would be needed to completely rule out the presence of amorphous YCl_3_ in the sample (the long relaxation time of ^89^Y spins, however, makes such experiments prohibitively time consuming). Non-stoichiometry will also lead to a range of bond angles and bond lengths around Na nuclei in the structure, which could partially account for peak broadening in the Na_3_YCl_6_ spectrum. Additional NMR experiments are underway, as well as first principles calculations of NMR parameters, to fully assign the data presented here.

Previous NMR studies on solid NaYF_4_^30^ and on the NaF-YF_3_ molten system^31^ have shown that the ^23^Na chemical shift becomes more negative as the number of Y^3+^ ions in the second coordination shell increases. In stoichiometric Na_3_YCl_6_, the Na0 site shown in Fig. 3b has four YCl_6_ neighboring octahedra, two of which are edge-sharing with the Y nucleus at a distance of 4.0–4.2 Ǻ from the central Na, and two of which are corner-sharing with Y-Na distances of 4.3 and 4.9 Ǻ. In contrast, the Na1 site has six corner-sharing YCl_6_ neighboring octahedra, with Y being 5.0–5.1 Ǻ away from the central Na. The presence of non-stoichiometry or disorder on the cation lattice will reduce the number of Y in the vicinity of the central Na and more strongly affect the chemical shift of Na0 (fewer Y^3+^ neighbors that are closer to the central Na) compared to that of Na1 (larger number of Y^3+^ neighbors further away from the central Na). It will also lead to additional ^23^Na NMR signals at more positive ppm values. With this in mind, we tentatively assign the most intense ^23^Na resonance at −8.1 ppm to Na nuclei in locally stoichiometric Na0 sites, the resonance at 3.6 p.p.m. to locally stoichiometric Na1 sites and the lower intensity peaks in-between these two resonances to Na nuclei in distorted and/or Y-deficient Na0 and Na1 sites. This assignment is consistent with the fact that there are twice as many Na0 sites than Na1 sites in the structure, such that the most populated Na local environment is expected to be the locally stoichiometric Na0 site.

Interestingly, while the substitution of Y by Zr is expected to vastly increase the number of possible Na local environments, the ^23^Na NMR spectra collected on the mixed Y/Zr samples exhibit either a single major resonance (as for the *x* = 0.25 sample) or two dominant signals (as for the x = 0.5 and 0.75 samples). In NMR, if two or more environments are in fast chemical exchange on the experimental timescale, their signals coalesce into a single peak at the weighted average of their resonant frequencies. Notably, the exchange rate at which the signals coalesce depends on the chemical shift separation of the individual resonances for the exchanging sites. Hence, the presence of few resonances in the spectra collected on the *x* = 0.25, 0.5 and 0.75 compounds indicate fast Na^+^ diffusion in these structures, consistent with the ionic conductivity measurements presented earlier. Given that all three compounds exhibit relatively similar Na diffusion properties, the fact that a single peak is observed in the *x* = 0.25 sample while two broader peaks are observed for the *x* = 0.5 and 0.75 samples may stem from a smaller distribution of Na local environments in the former, leading to chemical shifts that are closer together than in the *x* = 0.5 and 0.75 compounds. Finally, the spectrum collected on NZC indicates the presence of at least three distinct Na sites in the structure, not including the NaCl peak at 7.2 ppm. Since the ideal NZC structure contains a single Na environment, the three distinct ^23^Na resonances could indicate non-stoichiometry or local structural disorder. In addition, Na^+^ diffusion in this compound is too slow to lead to coalescence of the ^23^Na resonances of the exchanging sites.

### Mechanism for enhanced conductivity

**Fig. 4:**
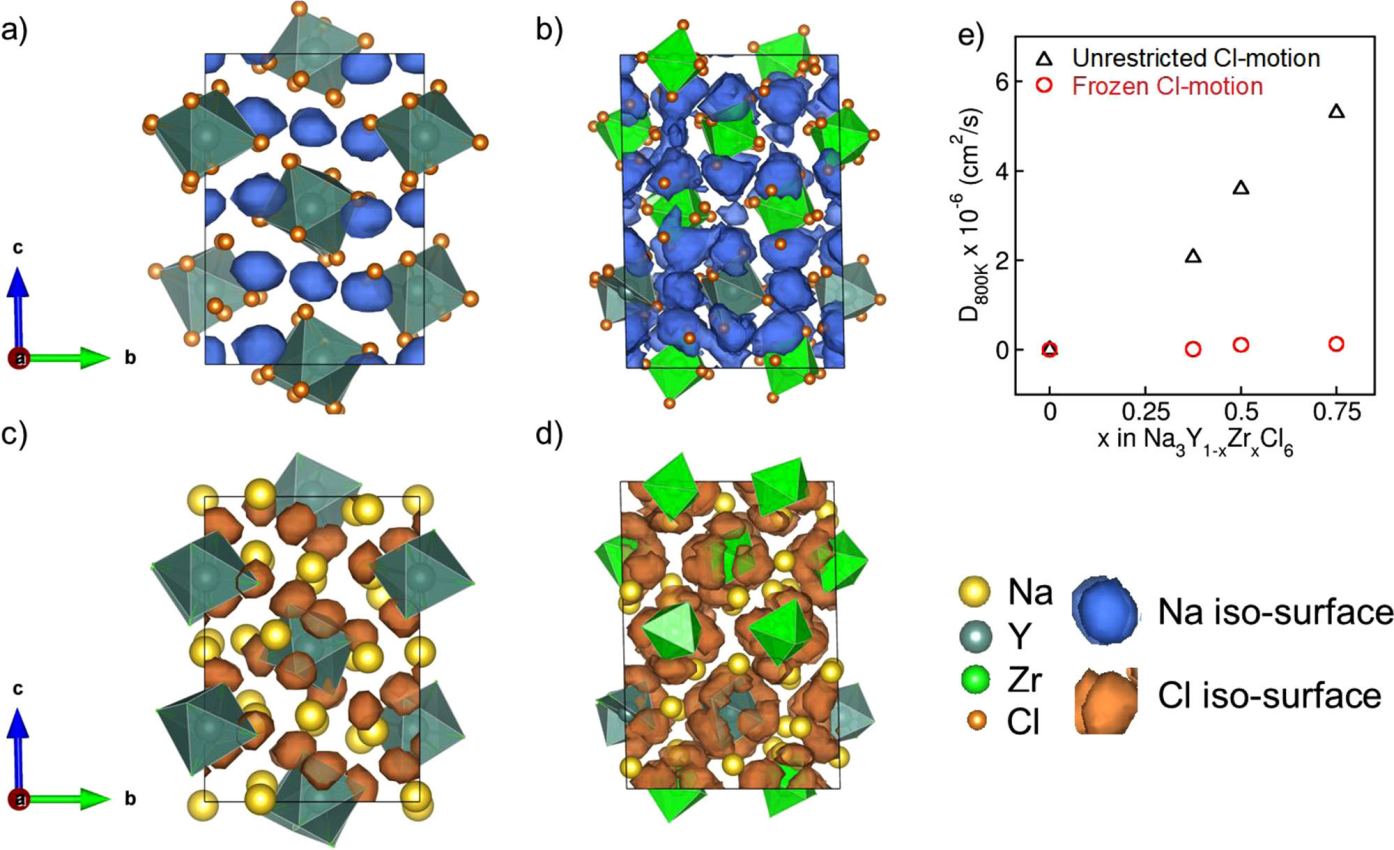
Effect of octahedra rotation on Na^+^ diffusivity. Plots of the probability density (isosurface value = 5 × 10^−4^) of **a** Na^+^ in Na_3_YCl_6_, **b** Na^+^ in Na_2.25_Y_0.25_Z_0.75_Cl_6_, **c** Cl^−^ in Na_3_YCl_6_ and **d** Cl^−^ in Na_2.25_Y_0.25_Z_0.75_Cl_6_, over 100 ps of AIMD simulations at 600 K. The motion of Na^+^ and Cl^−^ in Na_3_YCl_6_ are relatively localized, while macroscopic Na^+^ diffusion with (Zr/Y)Cl_6_ octahedral rotation are observed in Na_2.25_Y_0.25_Z_0.75_Cl_6_. **e** Na^+^ diffusivity at 800 K (D_800K_, in cm^2^/s) for varying Zr content in Na_3−*x*_Y_1−*x*_Zr_*x*_Cl_6_, compared with a selective dynamics simulation with Cl^−^ ions frozen in space, which shows negligible Na^+^ diffusivity.

To probe the origins of the greatly enhanced conductivity in NYZC*x*, we compared the probability distributions for both Na^+^ and Cl^−^ in NYC and NYZC0.75 extracted from 100 ps of AIMD trajectory simulations at 600 K. In NYC, the Na^+^ trajectories (Fig. 4a) indicate mostly local Na^+^ motion with little long-range transport, consistent with the observed low Na^+^ conductivity. In contrast, fast macroscopic 3D Na^+^ diffusion is observed for NYZC (Fig. 4b). Interestingly, there are substantial differences in the trajectories of the anion framework as well. While the Cl^−^ remain relatively static in NYC even at these elevated temperatures (Fig. 4c), substantial Cl^−^ motion, corresponding to YCl_6_^3−^/ZrCl_6_^2−^ octahedra rotations, are observed in NYZC0.75 (Fig. 4d). While similar behavior has been observed in lithium superionic conductors containing borohydride anions B_10_H_10_^2−^ ^32^, B_12_H_12_^2−^ ^33^, and BH_4_^−^ ^34^, and the sulfides β-Li_3_PS_4_ and Li_3.25_Si_0.25_P_0.75_S_4_^35^, this is the first observed instance of rotational motion of Cl^−^ in a halide single ion conductor. It is important to note that while there is polyanionic rotation, there is no signature for Zr(Y)-Cl bond breaking (Supplementary Fig. 9).

To investigate the effects of octahedral rotations and lattice volume on Na^+^ conductivities, two gedankenexperiments were performed with NYZC0.75 where YCl_6_^3−^/ZrCl_6_^2−^ octahedra were either frozen in their initial positions or the NYZC0.75 lattice was constrained to the lattice volume of NYC. At 800 K, NYZC*x* with frozen YCl_6_^3−^/ZrCl_6_^2−^ octahedra does not exhibit significantly higher Na^+^ diffusivity compared to NYC despite the presence of Na^+^ vacancies and an increased unit cell volume (Fig. 4e). Nevertheless, an increased cell volume due to Zr^4+^ doping is necessary for YCl_6_^3−^/ZrCl_6_^2−^ octahedral rotations to occur; when NYZC0.75 is constrained to have the same unit cell volume as NYC, the magnitude of the octahedral rotations is greatly reduced (Supplementary Fig. 10), as is ionic conductivity. These AIMD calculations therefore indicate that an increase in cell volume or in octahedral motion significantly improves Na^+^ conductivity - both effects are closely related and most likely responsible for enhanced Na^+^ diffusion kinetics in NYZC0.75, as compared to NYC.

In addition, an analysis of Na^+^ motion in NYZC0.75 was carried out using the trajectories from the ML-IAP NpT MD simulations at 500 K and 550 K, i.e., below and above the transition point for the two linear regimes in Fig. 1d, respectively. It was found that the Na^+^ diffusion topology changes from being quasi-2D to being 3D at the transition temperature, accompanied by a sharp increase in the degree of YCl_6_^3-^/ZrCl_6_^2-^ octahedra rotation (Supplementary Fig. 11). We may therefore surmise that the much lower barriers for Na^+^ diffusion in the high-temperature regime compared to the low-temperature regime is due to the activation of additional rotational modes and diffusion pathways above the transition temperature. These results highlight the cooperative interplay between increased lattice volume^36^ and octahedral rotations in enhancing the long-range Na^+^ conductivity in this framework.

### Cathode composite for a long cycle-life solid-state sodium battery

**Fig. 5:**
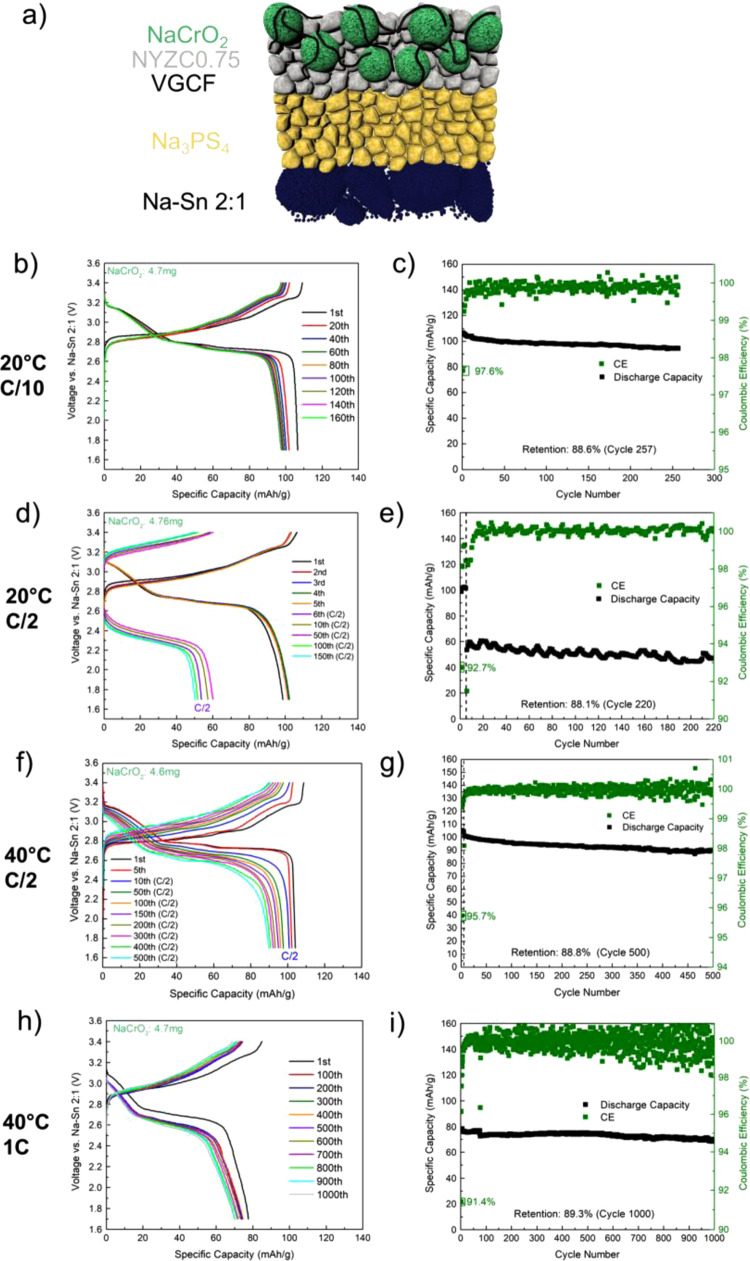
Electrochemical performance of the NYZC0.75 SSSBs. **a** Cell schematic. Voltage profile and specific capacity as a function of cycle number of this cell configuration, respectively, running at: **b**–**c** 20 °C and C/10, **d**–**e** 20 °C and C/10 for the first 5 cycles and subsequent cycling at C/2, **f**–**g** 40 °C and C/10 for the first 5 cycles and subsequent cycling at C/2, and **h**–**i** 40 °C and 1 C. In each case, the NYZC0.75 cells exhibit long-term cycling stability, with 89.3% capacity retention at 1000 cycles for the 40 °C 1 C cell.

Given the high cathodic stability and conductivity of NYZC0.75, cells comprising NYZC0.75 in a composite with the NaCrO_2_ cathode and Na_3_PS_4_ as the SE were constructed; a schematic is shown in Fig. 5a. For comparison, a control cell using Na_3_PS_4_ alone, without NYZC0.75, was also constructed (Supplementary Fig. 12a). At 20 °C at a rate of C/10 (Fig. 5b, c for NYZC0.75 and Supplementary Fig. 12b, c for NPS), it is evident that the first cycle Coulombic efficiency (CE) drastically increased in the NYZC0.75 cell (from 71.9% to 97.6%). This observed first cycle CE for the NYZC0.75 cell is the highest among those reported for Na ASSBs that use NaCrO_2_ as the cathode^5,37–39^. We believe that the high cathodic stability of NYZC0.75 protects the Na_3_PS_4_ SE from oxidation by NaCrO_2_, and in turn the Na_3_PS_4_ SE forms a stable passivating interface with the Na-Sn anode^7^. This is consistent with results from symmetric cell experiments carried out with NYZC*x* and NPS with the Na-Sn alloys Na_15_Sn_4_ (0.1 V vs. Na/Na^+^) and Na-Sn 2:1 (0.3 V vs. Na/Na^+^), as shown in Supplementary Fig. 13^40^. Based on the results, Na-Sn 2:1 was chosen for its stability with NPS.

**Fig. 6:**
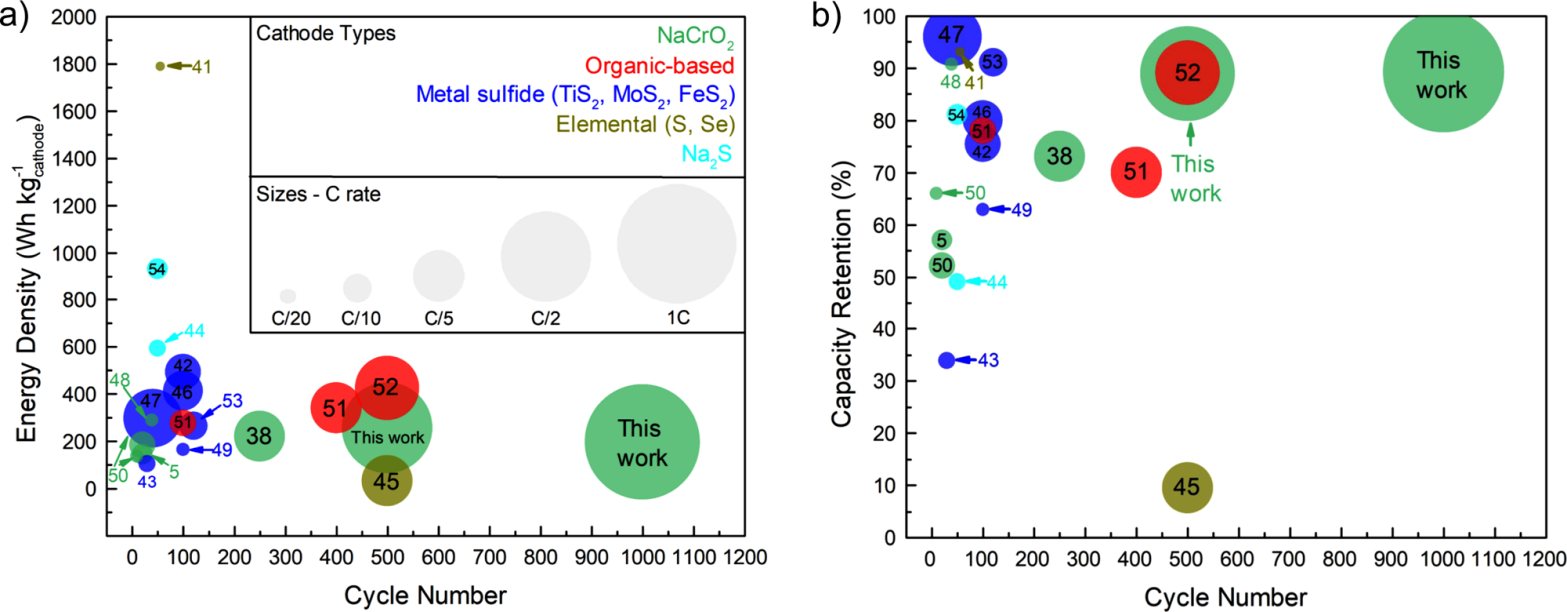
Comparison of SSSB performance metrics. **a** Gravimetric energy density (per mass of active material) plotted as a function of cycle number. **b** Capacity retention as a function of cycle number^5,38,41–54^. The cycling performance comparison highlights the compatibility and stability of the NaCrO_2_ + Na_2.25_Y_0.25_Zr_0.75_Cl_6_ + VGCF composite cathode.

To study the rate capability of the NYZC0.75 cell configuration, additional cells were constructed and tested at C/2 (after the first 5 cycles at C/10) at both 20 °C and 40 °C (Fig. 5d–g, respectively). At 20 °C, there is a noticeable drop in specific capacity (from 101 to 53.7 mAh/g) after switching to a rate of C/2. This is due to several reasons: one, the NPS layer is relatively thick (~800 µm) and the conductivity of NYZC0.75 is in the order of 10^−5^ S/cm. It is important to note that the cyclical behavior in Fig. 5e is due to temperature variations in the glovebox, as the cell was not inside a temperature-controlled chamber. At 40 °C, where the conductivity of NYZC0.75 is in the range of 1–2 × 10^−4^ S/cm, thus increasing the kinetics, the drop in capacity is negligible (from 104 to 101 mAh/g) when switching to a rate of C/2. For this particular SSSB, the average CE is 99.96%, which yields a capacity retention of 88% after 500 cycles. Furthermore, another NYZC0.75-NPS SSSB was constructed and cycled at 40 °C and at a rate of 1 C (Fig. 5h, i). Although there is a slight drop in capacity (78 mAh/g) compared to the cell cycled at 40 °C and C/2 (101 mAh/g), the cell cycled at 1 C lasted over 1000 cycles with a capacity retention of 89.3%, highlighting the superior stability of NYZC0.75 when paired with NaCrO_2_. To date, this is the highest capacity retention obtained for a SSSB, and Fig. 6 compares cycling performance metrics (gravimetric energy density per active mass, cycle life, capacity retention, rate, and cathode type) across various SSSB reports^5,38,41–54^. It is important to note that the cathode composite contains 39% of active material, NaCrO_2_, which is far from practical values. However, these results illustrate that a NYZC0.75-NaCrO2-VGCF cathode composite matrix exhibits stable electrochemical performance with prolonged cycling. Increasing the active mass loading is a necessary direction for future work.

Furthermore, an NPS control cell was also cycled 40 °C and C/10 (Supplementary Fig. 12d, e); the first cycle CE decreased from 71.9% (at 20 °C) to 62.4%, showing that NPS oxidation is exacerbated at 40 °C. This result contrasts with the demonstrated superior electrochemical stability of NYZC0.75 at 40 °C and high rates.

To investigate the effect of reducing the thickness of NPS, a modified cell design with a stainless steel (current collector) support was fabricated, such that the amount of NPS was reduced by half. Additional details can be found in Supplementary Note 4, and the rate capability test and EIS are shown in Supplementary Fig. 14. Based on these results, thinning the electrolyte layer (or reducing the amount of inactive material) and further optimization of the cell configuration is a promising avenue for future work.

To characterize the chemical environments in NPS and NYZC0.75 components after cycling, the SSSBs were disassembled to recover the composite cathodes and XPS measurements were conducted. Supplementary Fig. 15 compares the S *2p* and P *2p* binding energy regions of pristine NPS and the cycled NPS-containing composite cathode. Consistent with previous reports, when paired with an oxide cathode, NPS oxidizes to form elemental sulfur, other P_2_S_x_ compounds, and possibly compounds containing P-O bonds^21,55,56^. Supplementary Fig. 16 shows the Zr *3d* and Y *3d* bonds of pristine versus cycled NYZC0.75. Even with cells cycled at elevated temperatures or high rates, the Zr-Cl and Y-Cl bonds are retained in the composite cathode, confirming the electrochemical stability of NYZC0.75 when used with NaCrO_2_.

To evaluate the chemical stability, temperature-dependent XRD patterns were collected for 1:1 mixtures of NPS:NaCrO_2_ and NYZC0.75:NaCrO_2_ (Supplementary Fig. 17). In both the cases, no new additional peaks appeared, indicating no chemical reaction, even at temperatures as high as 220 °C. This is in accordance with Supplementary Table 2, where the reaction energy with NaCrO_2_ is low for both NPS (−0.18 eV/atom) and NYZC0.75 (−0.14 eV/atom). Thus, the observed superior cycling stability of NYZC0.75 arises from its intrinsic chemical stability in combination with its wide electrochemical window, whereas NPS is electrochemically unstable in the presence of NaCrO_2_ and undergoes oxidative decomposition during charging.

In summary, we reported on aliovalent substitution in the halide-based ionic crystal Na_3_YCl_6_, which leads to phases with enhanced Na^+^ conduction due to the presence of an interconnected network of Na^+^ diffusion channels (specifically, substituting Y^3+^ in Na_3_YCl_6_ with Zr^4+^ to form Na_3-*x*_Y_1-*x*_Zr_*x*_Cl_6_). Zr^4+^ substitution was found to increase the volume of the unit cell, which in turn enables polyanion rotation. The synergy between polyanionic rotation and increase in the effective mobile carrier concentration leads to a significant increase in the Na^+^ diffusivity upon Zr incorporation, which is absent in the parent Na_3_YCl_6_ compound. This was confirmed experimentally by an increase in the ionic conductivity by two orders of magnitude upon Zr substitution. Furthermore, the wide oxidative electrochemical window (up to 3.8 V) was retained after substitution, which proved to be beneficial when paired with a NaCrO_2_ cathode in a model SSSB. In this configuration, no electrochemical decomposition was observed, in contrast with a cell comprising Na_3_PS_4_ in the cathode composite, as revealed by XPS. At 40 °C and a rate of 1 C, the cell containing Na_2.25_Y_0.25_Zr_0.75_Cl_6_ was able to cycle over 1000 cycles with a capacity retention of 89.3%, the highest cycle life for a SSSB to date. Thus, further exploration of halide-based materials, especially in SSSBs, is a worthy area of continued investigation. This methodology of coupling computational and experimental evaluation, verification, and testing of material properties is an effective and necessary strategy toward finding compatible, long-lasting, and high-performing SSSB chemistries.

## Methods

### Structural relaxations and energy calculations

All density functional theory (DFT) calculations were performed using the projector augmented wave (PAW) approach as implemented in the VASP package^57,58^. The Perdew-Burke-Ernzerhof (PBE) generalized gradient approximation functional was used^59^. A plane-wave cut-off of 520 eV was used for DFT relaxations and energy calculations, consistent with the settings used in the Materials Project database^60^. All input file generation and post-processing analysis were performed using Pymatgen and pymatgen-diffusion packages^61^. Spin-polarized DFT calculations revealed that the net magnetization is nearly zero (≈ 0.01) for NYZC since both Y^3+^ and Zr^4+^ exhibit diamagnetic properties (i.e. noble gas configuration). In addition, NYZC, unlike the electrode, does not undergo any redox process. Thus, the net magnetization for NYZC should always be zero, therefore calculations were carried out without spin polarization.

The pre-relaxed structures of Na_3_YCl_6_ (mp-31362) and Na_3_YBr_6_ (mp-29080) were extracted from the Materials Project (MP) database^62,63^. The corresponding ICSD^64^ IDs are #59886 and #82355, respectively. Aliovalent substitution on the Y^3+^ sites with charge compensation by Na^+^ vacancies were performed to generate Na_3-(*z*-3)*x*_Y_1-*x*_M^z+^_*x*_Cl_6_ (M = Ti^4+^, Zr^4+^, Hf^4+^, Ta^5+^) structures. DFT calculations were performed on all symmetrically distinct orderings of Y/M and Na/vacancies to identify the lowest energy structure. Candidate structures for the Na_2_ZrCl_6_ phase were obtained by performing ionic substitutions of all structures in MP database matching the formula of A_2_MX_6_. All candidate structures were fully relaxed using DFT prior to calculating their energies. The computed XRD pattern of the lowest energy candidate was successfully matched to the experimental XRD pattern.

Other than the target phases of interest, the pre-computed energies of all other structures in the Na-Y-Zr-Cl phase space were obtained from the Materials Project database and used in the calculation of the energy above hull (E_hull_), electrochemical stability window, and the interfacial reaction products following the methodologies established in prior publications^21,65^.

Topological analysis of the framework chemistries was performed using Zeo ++, an open source topological analysis package^66^. The quantity of interest is the largest included sphere radius along the free sphere path R_inc_. This gives an estimate of the diffusion channel size which is associated with the ionic conductivity of the material.

### Ab initio molecular dynamics

Non-spin polarized ab initio molecular dynamics (AIMD) simulations were carried out in the NVT ensemble. From the spin-polarized DFT calculations, the net magnetization is 0 for all ions, which supports the use of non-spin-polarized calculations for AIMD. A plane-wave energy cutoff of 280 eV, supercells with the minimum dimension larger than 10 Å and a minimal Γ-centered 1 × 1 × 1 k-mesh were used. The time step was set to 2 fs. Simulations were carried out at several temperatures between 500 K and 1200 K and the corresponding diffusivities were extracted using the Nernst-Einstein relationship from the slope of the plot of the mean square displacement of Na ions with time.

### Machine learning interatomic potential and molecular dynamic simulations

The moment tensor potential (MTP)^23–25^ for NYZC0.75 was developed using the open-source Materials Machine Learning (maml) Python package. The training data comprises 800 snapshots extracted at 400 fs intervals from AIMD NVT simulations at 600 K, 800 K, 1000 K, and 1200 K. Static DFT calculations were then performed to obtain accurate energies and forces. A training:test split of 90:10 was used to train the machine learning model. The MTP cutoff radius and the maximum level of basis functions, lev_max_ were chosen to be 5.0 Å and 14, respectively. The mean absolute error (MAE) on the energies and forces were 1 meV atom^−1^ and 63.5 meV Å^−1^, respectively (Supplementary Figure 18 and Supplementary Table 5). NPT MD simulations using the MTP were carried out using LAMMPS^67^. The simulation time was at a least amount of 10 ns with a 2 fs time step. A 4 × 4 × 4 supercell of the NYZC0.75 with 592 atoms was used.

### Material synthesis

All fabrication processes were conducted in an Ar-filled glovebox (mBraun 200B, H_2_O ppm <0.5, O_2_ ppm < 1), unless otherwise noted.

Stoichiometric amounts of the precursors NaCl (>99%, Sigma Aldrich), YCl_3_, (99.9%, Sigma Aldrich) were hand-mixed in a mortar and pestle for 10 min and the powder mixture was placed in a 50 mL ZrO_2_ ball mill jar (Retsch Emax) with eleven 10 mm-diameter Y-ZrO_2_ milling balls. The mixture was milled for 2 h at 500 r.p.m. The material was extracted from the jars in the glovebox, pelletized at a pressure of 370 MPa with a 13 mm pellet die (Carver), loaded into a quartz tube, flame sealed, and heated in a box furnace (Lindberg Blue M) at 500 °C for 24 h. To homogenize the material, the material was ball milled again after heat treatment using 88 5 mm diameter Y-ZrO_2_ milling balls for a duration of 4 h. The material was extracted and stored in the glovebox before further testing.

For the Zr substituted compounds, the same procedure was conducted with the addition of ZrCl_4_ (99.99%, Sigma Aldrich) as a third precursor, and the reagent ratios adjusted according to stoichiometry.

### Characterization—XRD

Powder samples were loaded into 0.5 mm-diameter boron-rich capillary tubes (Charles Supper). The tube opening was capped with clay and wrapped in paraffin film before it was brought outside of the glovebox to be flame-sealed with a butane torch. The samples were measured on a Bruker Kappa goniometer equipped with a Bruker Vantec 500 detector. The sample was placed in the Bragg−Brentano θ − θ configuration and the Debye−Scherrer method was used for measurements. XRD data was collected using Cu Kα radiation at 45 kV and 50 mA, over a 2θ range of 5 − 90° with a step size of 0.01°. Rietveld refinement was carried out with the FullProf software suite.

For temperature-dependent capillary XRD, the capillary tubes were heated at a rate of 5 °C/min and held at the target temperature for one hour before the XRD measurement was taken.

For Synchrotron XRD, the samples were prepared by loading the powders into polyimide tubes in the glovebox and were subsequently sealed with epoxy. Measurements were carried out at Beamline 28-ID-1 at NSLS-II.

### Characterization—electrochemical

75 mg of powder was pressed at 370 MPa into a 10 mm polyether ether ketone (PEEK die) using two titanium plungers and the dimensions were measured with calipers. The relative density of the pellets was determined by comparing the measured dimensions with the XRD results. On both sides of the pellet, acetylene black (AB) was added for better contact with the current collectors; once added, the AB was also pressed at 370 MPa using the titanium plungers. The cell configuration was secured into a cell holder and connected to a Solartron 1260 impedance analyzer. Impedance measurements were taken with an applied AC potential of 30 mV over a frequency range of 1 MHz to 1 Hz. Temperature-dependent EIS measurements were also conducted within the glovebox; the sample was heated from 20 °C to 100 °C and EIS measurements were recorded at every 20 °C increment. Measurements were taken only after the sample was held at the target temperature for over an hour to allow for equilibration. The heating rate was 2 °C/min. The activation energy (E_a_) was calculated from the slope of the resulting Arrhenius plot.

DC polarization was also conducted by the Solartron 1260 impedance analyzer. The cell setup was similar as before; the powder was pressed at 370 MPa into a 10 mm PEEK die using two titanium plungers and subsequently secured into a cell holder. The applied DC potential was 50 mV and the current response was measured over time.

The model SSSB is composed of NaCrO_2_ as the positive electrode, Na-Sn (2:1) as the negative electrode, and Na_3_PS_4_ as the electrolyte. The positive electrode is mixed into a composite with a weight ratio of 11:16:1 of NaCrO_2_: Na_3_PS_4_: VGCF (VGCF from Sigma Aldrich; length 20–200 μm, <100 ppm iron, average diameter: 130 nm, average specific surface area: 24 m^2^ g^−1^). The battery is fabricated through mechanical pressing; 75 mg of Na_3_PS_4_ powder is pressed first at 370 MPa, then about 12 mg of the composite NaCrO_2_ powder is placed on one side of the Na_3_PS_4_ pellet and pressed at the same pressure, and finally on the opposite side of the Na_3_PS_4_, an excess of Na-Sn 2:1 alloy (35 mg) is pressed at the same pressure. After securing the cell in a cell holder, the electrical leads were connected to an electrochemical cycler (Landhe). For a rate of C/10, the current density used was 64 µA cm^−2^.

To incorporate Na_2.25_Y_0.25_Zr_0.75_Cl_6_ (NYZC0.75) into the model SSSB, NYZC0.75 replaced Na_3_PS_4_ in the composite cathode (still hand-mixed with the same 11:16:1 ratio). For cells cycled at 40 °C, the cell assemblies were placed into a compact box furnace (MTI KSI-1100X) within the Ar-filled glovebox. Current densities ranged from 64 µA cm^−2^ (C/10) to 640 µA cm^−2^ (1 C).

After cycling, the cell was disassembled to characterize any material changes.

### Characterization—^23^Na solid-state NMR

All ^23^Na solid-state NMR experiments were performed on a 3.2 mm HX probe on a Bruker Avance III Ultrashield Plus 800 MHz (18.8 T) NMR spectrometer. A 1 M NaCl aqueous solution, with a reported ^23^Na chemical shift of 0.04 ppm^68^ was used as reference sample to calibrate the chemical shift scale.

^23^Na NMR spectra were collected on NYZC*x* (*x* = 0, 0.25, 0.5, 0.75, 1) compounds with a simple pulse and acquire (zg) sequence and at a magic angle spinning (MAS) rate of 12 kHz as no spectral enhancement was observed at higher spinning speeds. The solid electrolyte samples were packed inside 3.2 mm sapphire rotors in an Ar-filled glovebox to avoid contamination with air or moisture. In addition, a flow of N_2_ gas was used to spin the samples, providing an inert atmosphere during ^23^Na NMR signal acquisition.

Due to the quadrupolar nature of ^23^Na nuclei (I = 3/2), the calibrated pulse durations differed for the various ^23^Na local environments in the samples. Hence, all spectra were obtained using a 30° radiofrequency (RF) excitation pulse in lieu of a standard 90° pulse angle to uniformly excite all ^23^Na spins in the sample and provide internally quantitative ^23^Na NMR spectra. The quantitative nature of the so-obtained spectra was confirmed by collecting data using a pulse angle as low as 5°, which showed no change in the relative amounts of each resonance compared to the 30° pulse data. The power level used for all measurements was 100 W (~93 kHz) with a 90° pulse duration of around 2.7 μs, therefore, a 30° pulse duration of either 0.9 or 0.95 μs was used depending on the optimization of each sample. A 30 s delay was applied before each scan when signal averaging in order to allow full relaxation, where the relaxation times of these samples are 2 s or below.

### Characterization—XPS

The powders were adhered onto a small metallic sample stub (Shimadzu) with carbon tape. The metallic stub was secured into a metallic canister and sealed inside the glovebox with clamps.

The metallic canister was placed into a N_2_ glovebox that is attached to the XPS tool (Kratos Axis Supra), where the sample can be transferred into the analysis chamber without any exposure to ambient air. All measurements were taken using 15 kV Al Kα radiation at a chamber pressure less than 5 × 10^−8^ torr. For the wide survey scans, a pass energy of 160 eV and a dwell time of 100 ms was used, but for specific element regions, a pass energy of 20 eV, a dwell time of 300 ms, and a step size of 0.05 eV was used. The charge neutralizer was enabled during all the measurements. Data calibration and analysis were conducted using the CasaXPS software, and all region spectra were calibrated using the C 1 s peak.

### Characterization—focused ion beam (FIB)

The Na_3-*x*_Y_1-*x*_Zr_*x*_Cl_6_ powders were pressed into a 10mm-diameter pellet at 370 MPa. The pellet was extracted and then mounted onto a SEM sample stage (Ted Pella) and transferred into the FEI Scios DualBeam FIB/SEM using the air-sensitive holder to avoid any ambient air exposure. Cross sections were milled using the FIB and SEM images were taken of the samples.

## Supplementary information

Supplementary Information

## Data Availability

The datasets generated and/or analyzed during the current study are available from the corresponding author on reasonable request. The X-ray crystallographic coordinates for the structures reported in this study, Na_3_YCl_6_ and Na_2_ZrCl_6_, have been deposited at the Cambridge Crystallographic Data Centre (CCDC), under deposition numbers 2057626-2057627. These data can be obtained free of charge from The Cambridge Crystallographic Data Centre via www.ccdc.cam.ac.uk/data_request/cif.

## Code availability

DFT calculations and AIMD simulations were carried out using the open-source Python Materials Genomics (pymatgen) package, which is available at http://pymatgen.org. The moment tensor potential was fitted using the open-source materials machine learning (maml) package, which is available at https://github.com/materialsvirtuallab/maml.
