# Supplementary material for: A stable cathode-solid electrolyte composite for high-voltage, long-cycle-life solid-state sodium-ion batteries
Source: Nat Commun. 2021 Feb 23;12:1256. doi: 10.1038/s41467-021-21488-7 (PMC7902639; doi:10.1038/s41467-021-21488-7)
Supplement: Supplementary file 1 — Supplementary Information [file 41467_2021_21488_MOESM1_ESM.pdf]

**Supplementary Information**

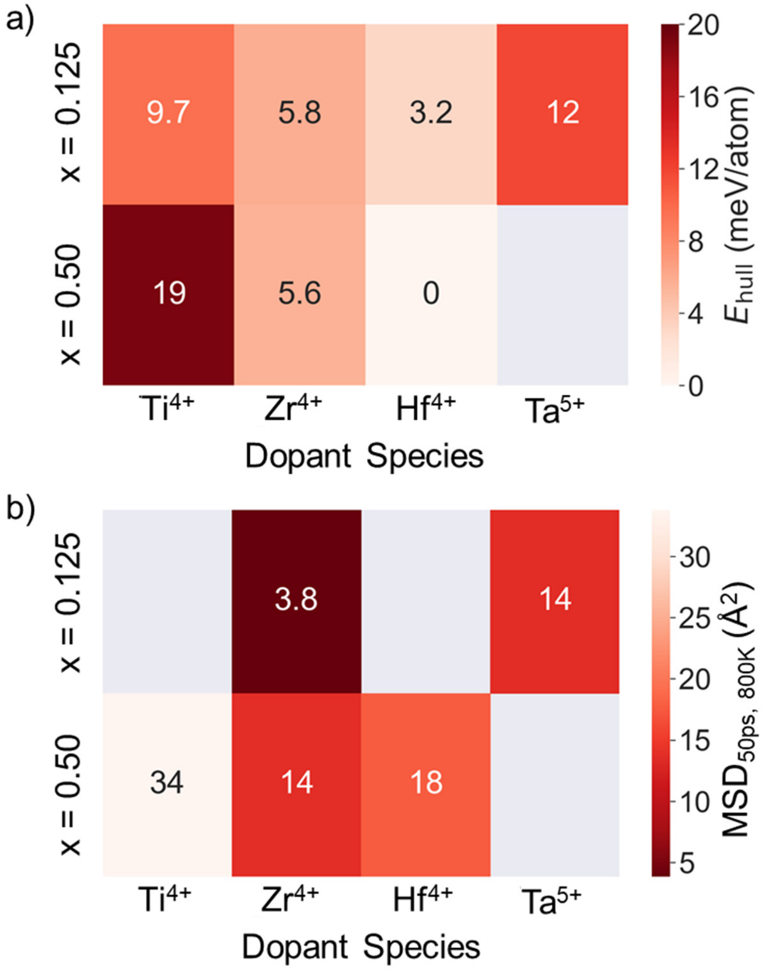


**Supplementary Figure 1 | Phase stability and diffusivity of substituted $Na_3YCl_6$ compounds. a**, Calculated energy above hull ($E_{hull}$) of $Na_{3-(z-3)x}Y_{1-x}M^{z+}{}_xCl_6$ ($M^{z+}$ = $Ti^{4+}$, $Zr^{4+}$, $Hf^{4+}$ , $x$ = 0.125, 0.50; $M^{z+}$ = $Ta^{5+}$, $x$ = 0.125). **b**, Mean squared displacement of $Na^+$ for a 50 ps time scale at 800 K ($MSD_{50ps,800K}$) of $Na_{3-(z-3)x}Y_{1-x}M^{z+}{}_xCl_6$ ($M^{z+}$ = $Zr^{4+}$, $Ta^{5+}$, $x$ = 0.125; $M^{z+}$ = $Ti^{4+}$, $Zr^{4+}$, $Hf^{4+}$, $x$ = 0.50). Gray regions indicate either an unstable compound (due to significant $Na^+$ loss for $Ta^{5+}$ at $x$ = 0.50) or in the case of MSD screening, preferentially conducted for the higher dopant concentrations for $Ti^{4+}$ and $Hf^{4+}$ (stable at $x$ = 0.50).

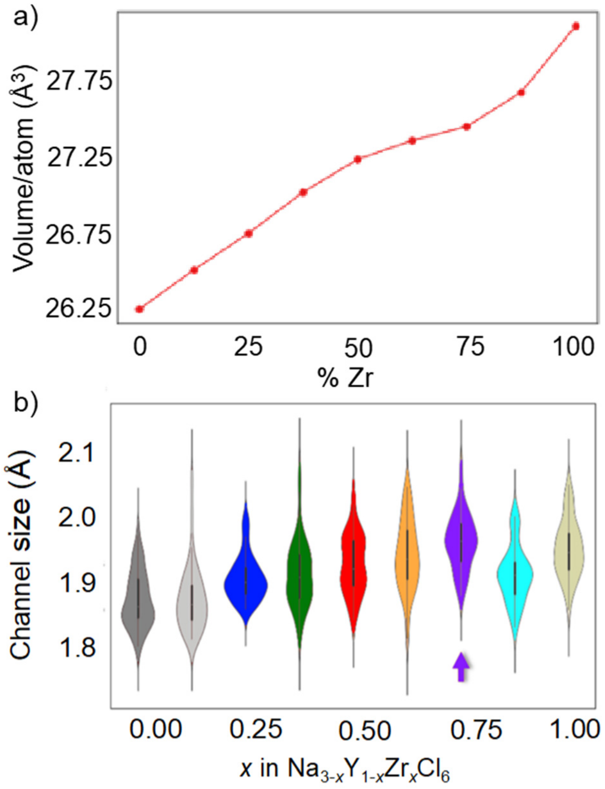


**Supplementary Figure 2 | Cell Dimensions of $Na_{3\text{-}x}Y_{1\text{-}x}Zr_xCl_6$.** **a**, Volume per atom and **b**, channel size changes with $x$ in $Na_{3\text{-}x}Y_{1\text{-}x}Zr_xCl_6$. The channel size was computed using a Voronoi-based algorithm. While the volume per atom increases monotonically with $x$, the channel size has a maximum at $x = 0.75$, which also corresponds to the composition with maximum $Na^+$ conductivity.

**Supplementary Table 1 | Comparison among $Na_3YBr_6$, $Na_3YCl_6$, and Zr-substituted $Na_3YCl_6$ ($Na_{2.5}Y_{0.5}Zr_{0.5}Cl_6$).** The crystal structure, thermodynamic stability ($E_{hull}$), $Na^+$ diffusion channel size, electronic band gap, and electrochemical stability window (EC window) are tabulated. The mean squared displacement of $Na^+$ for a 50 ps time scale at 800 K ($MSD_{50ps,\ 800K}$) is negligible for $Na_3YCl_6$ (NYC) and $Na_3YBr_6$ (NYB), implying that NYC and NYB have a rigid structure with little to no $Na^+$ diffusivity.

| Formula | **$Na_3YBr_6$** | **$Na_3YCl_6$** | **$Na_{2.5}Y_{0.5}Zr_{0.5}Cl_6$** |
|---|---|---|---|
| Crystal structure | 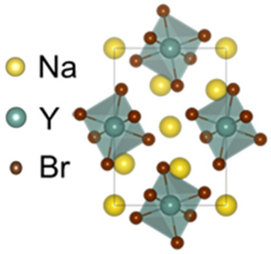  | 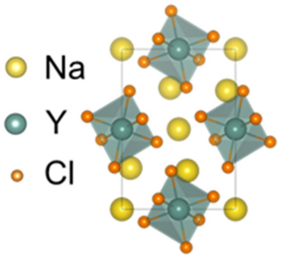  | 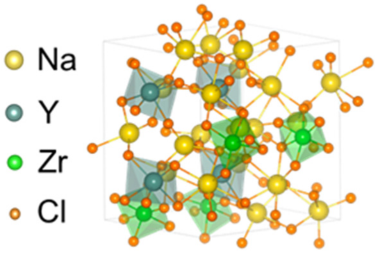  |
| Source | mp-29080 | mp-31362 | Substitution from $Na_3YCl_6$ |
| $E_{hull}$ (meV/atom) | 15 | 3 | 5.6 |
| Channel size (Å) | 1.94 | 1.81 | 1.88 |
| Bandgap (eV) | 4.25 | 5.18 | 5.65 |
| EC window (V) | 0.6-3.2 | 0.6-3.8 | 1.6-3.8 |
| $MSD_{50ps,\ 800K}$ (Å$^2$) | 1.0 | 0.8 | 13.7 |

**Supplementary Table 2 | Computed reaction Energies (with $NaCrO_2$ and Na metal) and the electrochemical windows of NYC, $Na_{2.25}Y_{0.25}Zr_{0.75}Cl_6$, and $Na_3PS_4$.**

| **System** | **Reaction energy w/ $NaCrO_2$ (eV/atom)** | **Reaction energy w/Na (eV/atom)** | **EC window (V)** |
|---|---|---|---|
| $Na_3YCl_6$ | -0.11 | -0.13 | 0.6-3.8 |
| $Na_{2.25}Y_{0.25}Zr_{0.75}Cl_6$ | -0.14 | -0.34 | 1.5-3.8 |
| Ref: c-$Na_3PS_4$ | -0.18 | -0.46 | 1.2-2.5 |

## Supplementary Note 1

The crystal structure of NYC was found to be monoclinic with the space group P 1 21/n 1, in good agreement with previous results.[1] Subsequently, the conductivity of NYC was measured

and the results are shown in Supplementary Figure 3b. At room temperature the ionic conductivity of NYC was determined to be 9.5 x $10^{-8}$ S/cm, several orders of magnitude lower than that of the Li counterpart, $Li_3YCl_6$ (1 x $10^{-4}$ S/cm).[2]

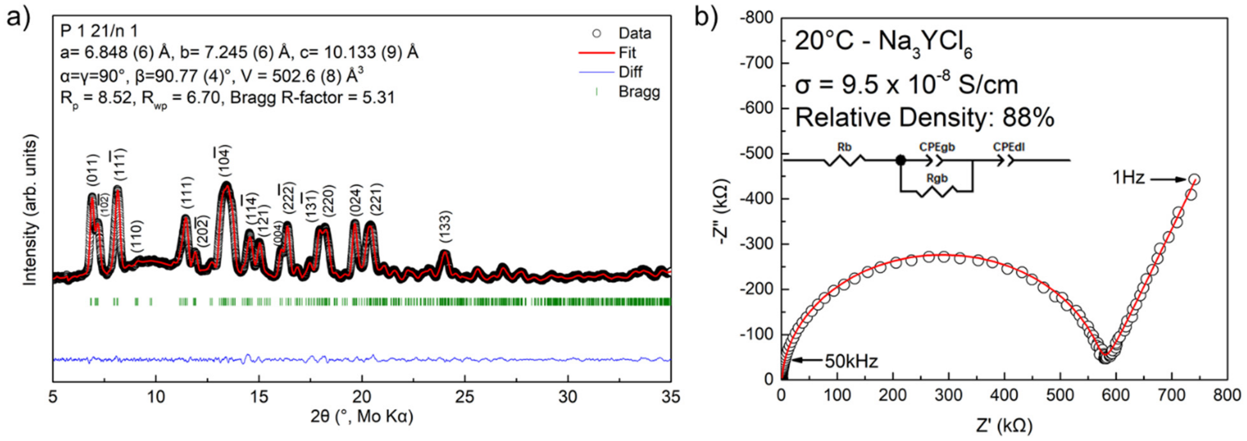


**Supplementary Figure 3 | Characterization of $Na_3YCl_6$.** **a**, Rietveld refinement result of the capillary XRD pattern of the as-synthesized $Na_3YCl_6$. The cell parameters and fitting parameters are in the insets. **b**, Room temperature Nyquist plot of $Na_3YCl_6$ and the equivalent circuit used for fitting; the conductivity was determined to be 9.5 x $10^{-8}$ S/cm.

**Supplementary Table 3 | Rietveld Refinement Results showing the Atomic Position, $B_{iso}$, and Occupancy values for $Na_3YCl_6$.**

| Na₃YCl₆ | | | | | |
|---|---|---|---|---|---|
| **Atom** | **x** | **y** | **z** | **$B_{iso}$** | **SOF** |
| Y | 0 | 0.5 | 0 | 3.1 (5) | 1 |
| Cl1 | 0.132 (3) | 0.567 (3) | 0.245 (4) | 3.5 (4) | 1 |
| Cl2 | 0.168 (4) | 0.801 (3) | 0.929 (3) | 3.5 (4) | 1 |
| Cl3 | 0.321 (4) | 0.325 (4) | 0.928 (3) | 3.5 (4) | 1 |
| Na0 | 0.519 (5) | 0.423 (3) | 0.245 (4) | 1.8 (7) | 1 |
| Na1 | 0.5 | 0 | 0 | 1.8 (7) | 1 |

## Supplementary Note 2

Structural relaxations were carried out using known structures with the formula $A_2MX_6$ from the Materials Project (MP) as well as ICSD. The results are shown in Supplementary Figure 4a-b; it was determined that $Na_2ZrCl_6$ was isostructural to $Na_2TiF_6$ and has the space group P -3 m 1.[3] This result is consistent with a recent report that described the structure on $Na_2ZrCl_6$.[4]

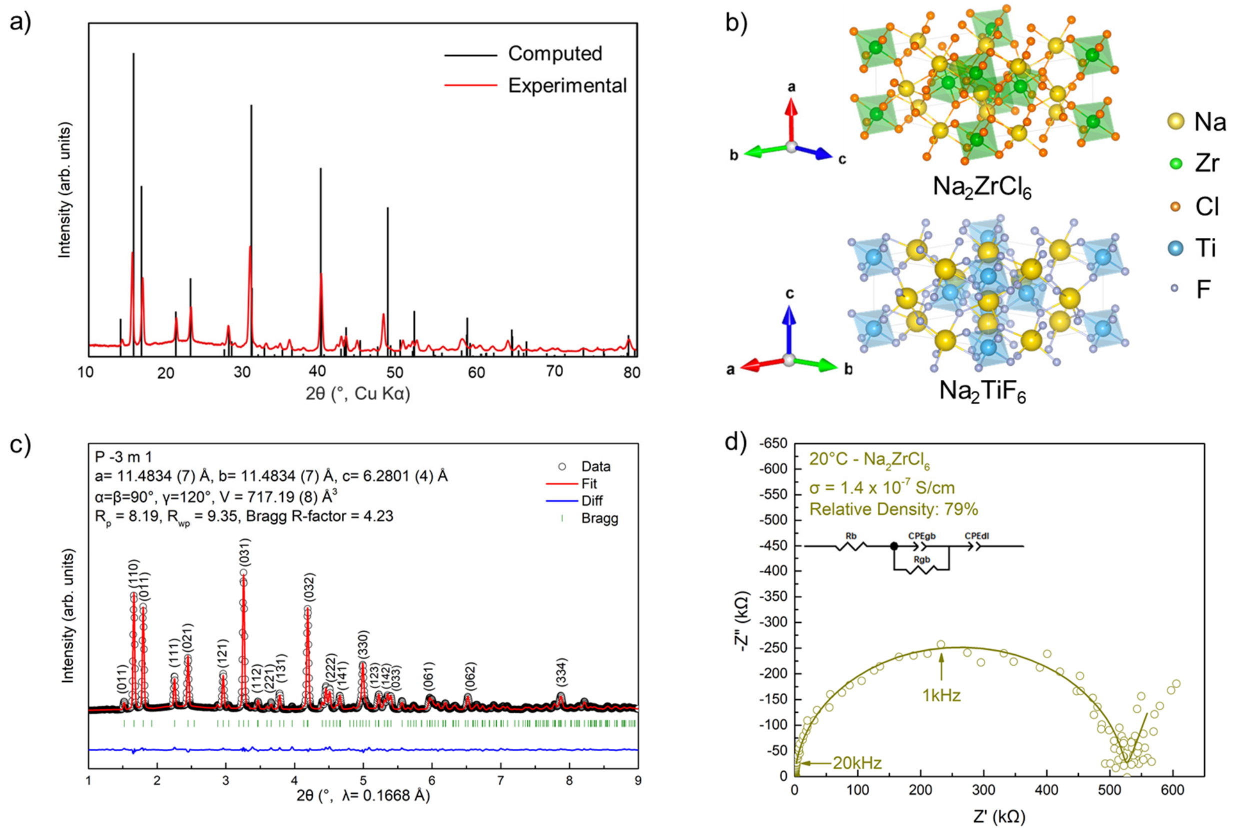


**Supplementary Figure 4 | Determination of the structure of $Na_2ZrCl_6$.** **a**, Computed XRD pattern of the determined $Na_2ZrCl_6$ structure overlaid with experimental XRD data for the post heat-treated $Na_2ZrCl_6$. **b**, (top) the structure of $Na_2ZrCl_6$, space group P -3 m 1, is isostructural to (bottom) $Na_2TiF_6$.[3] **c**, Rietveld

refinement result of the synchrotron XRD data of the post heat-treated $Na_2ZrCl_6$, in good agreement with the structural relaxation result. The cell parameters and fitting parameters are in the inset. **d**, Room temperature Nyquist plot and equivalent circuit fit for $Na_2ZrCl_6$.

**Supplementary Table 4 | Rietveld Refinement Results showing the Atomic Position, $B_{iso}$, and Occupancy values for $Na_2ZrCl_6$.**

| $Na_2ZrCl_6$ | | | | | |
|---|---|---|---|---|---|
| **Atom** | **x** | **y** | **z** | $B_{iso}$ | **SOF** |
| Zr1 | 0 | 0 | 0 | 1.20 (8) | 0.965 (5) |
| Zr2 | 0.3333 | 0.6667 | 0.4898 (10) | 1.20 (8) | 1 |
| Zr3 | 0 | 0 | 0.37 (2) | 1.20 (8) | 0.035 (2) |
| Cl1 | 0.1012 (11) | 0.8988 (11) | 0.2376 (9) | 3.68 (11) | 1 |
| Cl2 | 0.2316 (5) | 0.7684 (5) | 0.7039 (9) | 3.68 (11) | 1 |
| Cl3 | 0.4348 (11) | 0.5652 (11) | 0.2736 (9) | 3.68 (11) | 1 |
| Na1 | 0.3525 (8) | 0 | 0 | 3.0 (2) | 0.740 (8) |
| Na2 | 0.281 (3) | 0 | 0.5 | 3.0 (2) | 0.260 (8) |

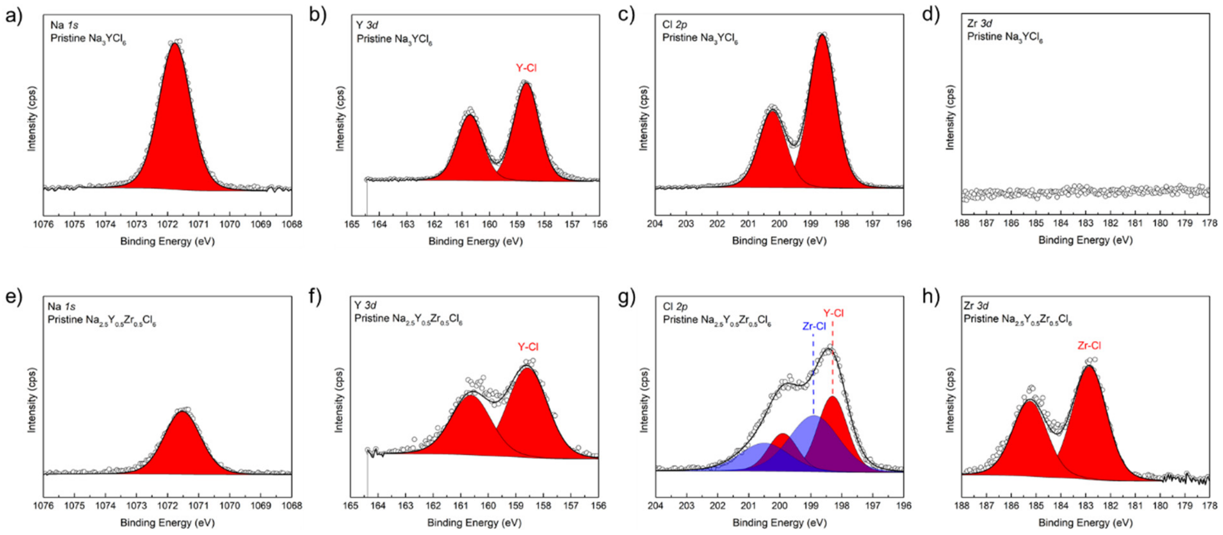


**Supplementary Figure 5 | XPS of the Na 1s, Y 3d, Cl 2p, and Zr 3d binding energy regions.** Shown for **a-d**, $Na_3YCl_6$ and **e-h**, $Na_{2.5}Y_{0.5}Zr_{0.5}Cl_6$, respectively. For $Na_3YCl_6$, the Cl *2p* binding energy region shows the Y-Cl bond energy signature and thus $YCl_6$ in the structure. For $Na_{2.5}Y_{0.5}Zr_{0.5}Cl_6$, there is an additional signature from the Zr-Cl bond as seen in the Cl *2p* and Zr *3d* binding energy regions, confirming the coexistence of both $YCl_6$ and $ZrCl_6$ units.[5]

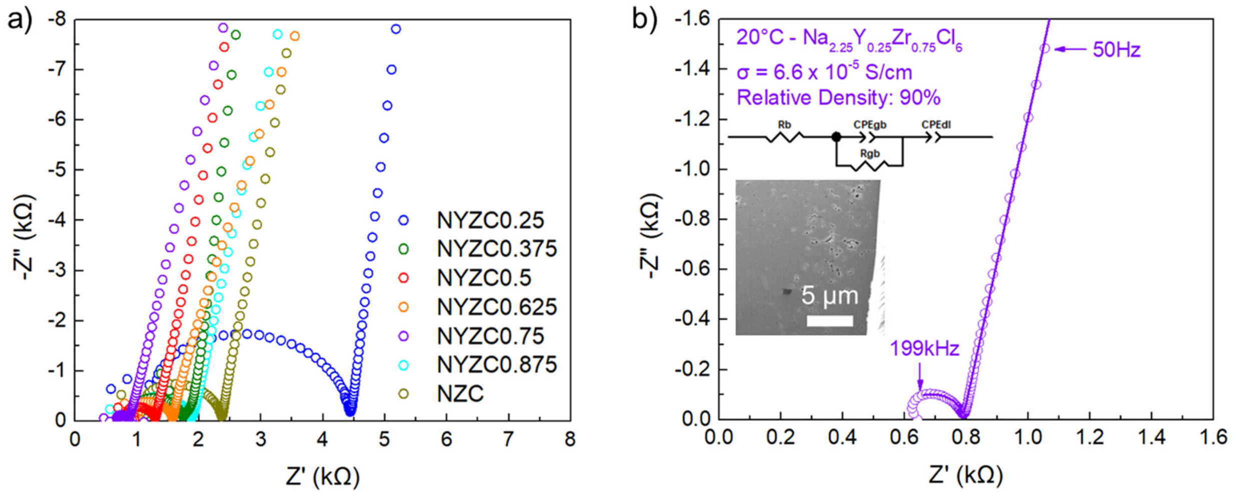


**Supplementary Figure 6 | Nyquist plots for $Na_{3-x}Y_{1-x}Zr_xCl_6$ (NYZC*x*). a**, The Nyquist plots from $x$ = 0.25 to $x$ = 1 are shown for scale. **b,** Equivalent circuit fit for $x$ = 0.75, the composition with the highest measured ionic conductivity. Inset: cross sectional image showing the dense morphology of the $Na_{3-x}Y_{1-x}Zr_xCl_6$ pellet.

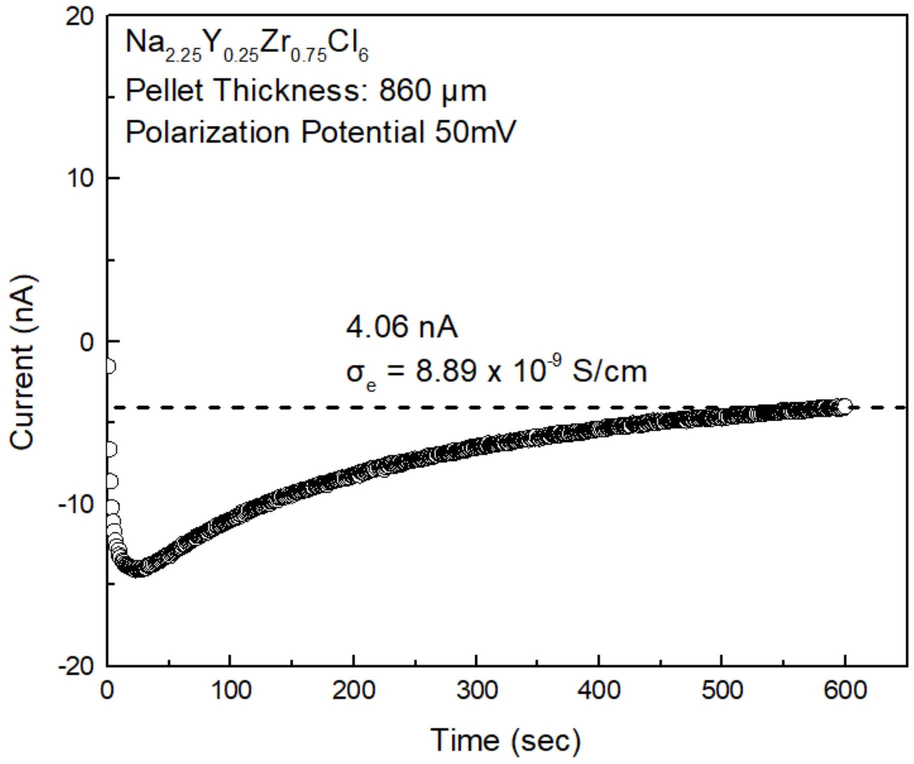


**Supplementary Figure 7 | Electronic Conductivity of $Na_{2.25}Y_{0.25}Zr_{0.75}Cl_6$.** DC polarization was conducted on a pellet of $Na_{2.25}Y_{0.25}Zr_{0.75}Cl_6$ to determine its electronic conductivity (8.89 x $10^{-9}$ S/cm, making it an electronic insulator). The applied potential was 50mV.

**Supplementary Note 3**

The $Na_{3-x}Y_{1-x}Zr_xCl_6$ compounds were all subjected to a synthesis procedure of mixing, heating at 500°C, quenching, and subsequent ball milling per the Methods section. This is because the conductivity of heat-treated $Na_{3-x}Y_{1-x}Zr_xCl_6$ is very low; in the case of heat-treated NYC before ball milling, the room temperature conductivity for NYC could not be measured as it is a poor $Na^+$ conductor. Similar to LYC, reducing the degree of crystallinity drastically increased the conductivity, so the ball milling procedure was used for every composition, and the ball-milled NYZC0.75 was the material incorporated into the ASSB.

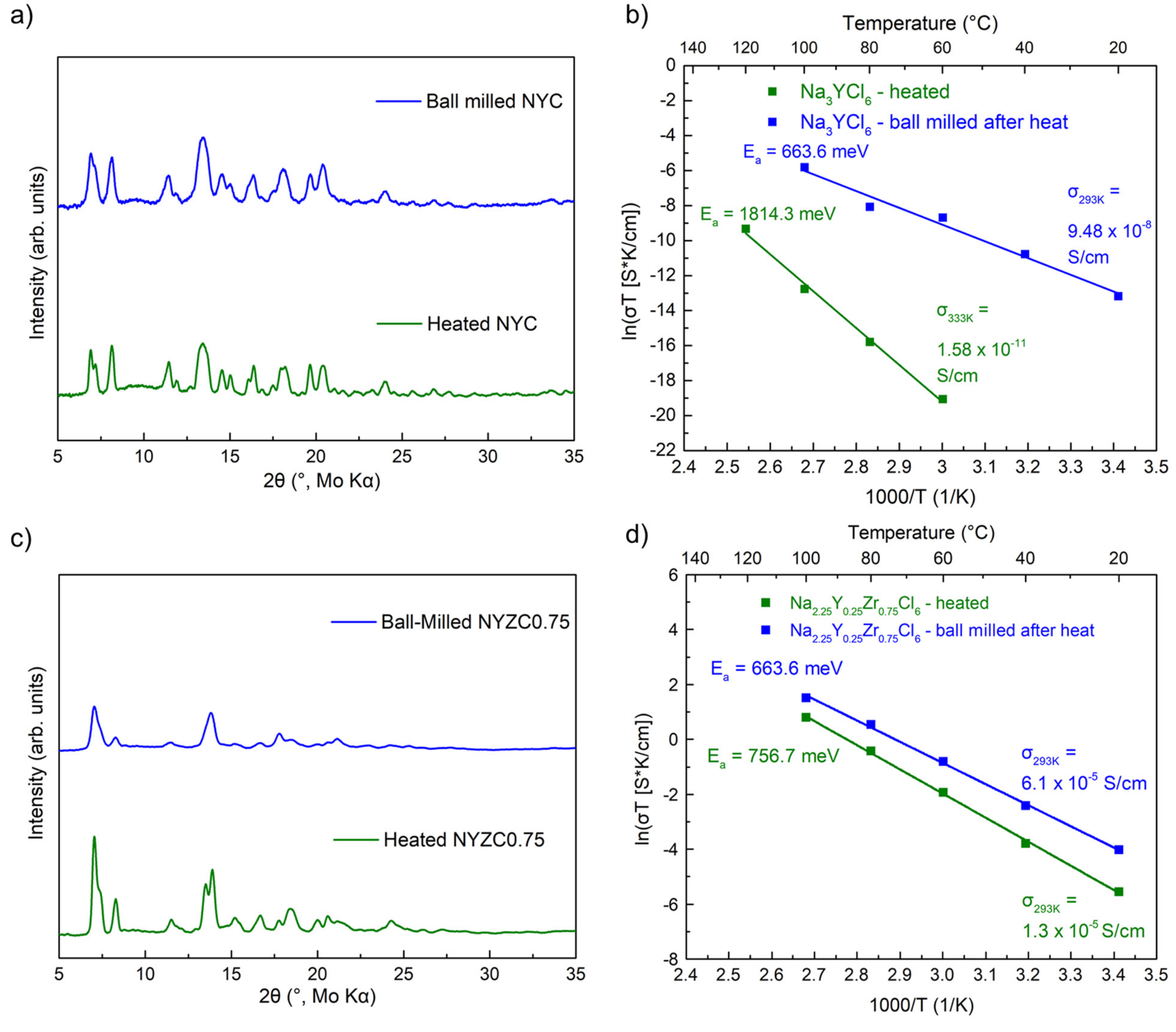


**Supplementary Figure 8 | XRD and Activation Energies before and after ball milling**. The XRD and Arrhenius plots, respectively, are shown before and after ball milling for **a**, **b**, $Na_3YCl_6$ and **c**, **d**, $Na_{2.25}Y_{0.25}Zr_{0.75}Cl_6$. In both cases, ball milling was found to lessen the degree of crystallinity (by decreasing peak intensities) and raise the conductivity.

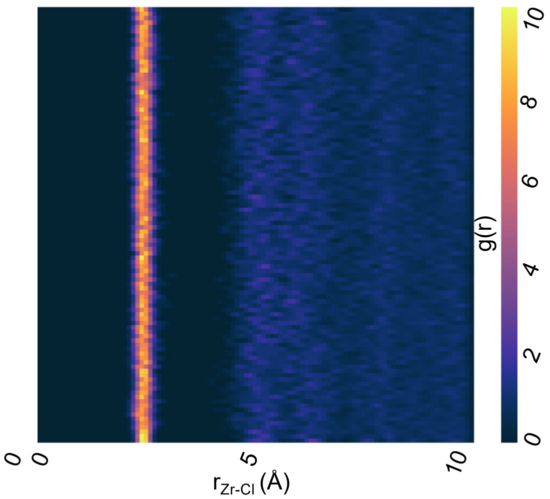


**Supplementary Figure 9 | Radial pair distribution function in $Na_{2.25}Y_{0.75}Zr_{0.25}Cl_6$.** Obtained from 100 ps AIMD simulation at 800 K. This result shows that the Zr-Cl bond does not break even though the $ZrCl_6$ unit undergoes rotational motion.

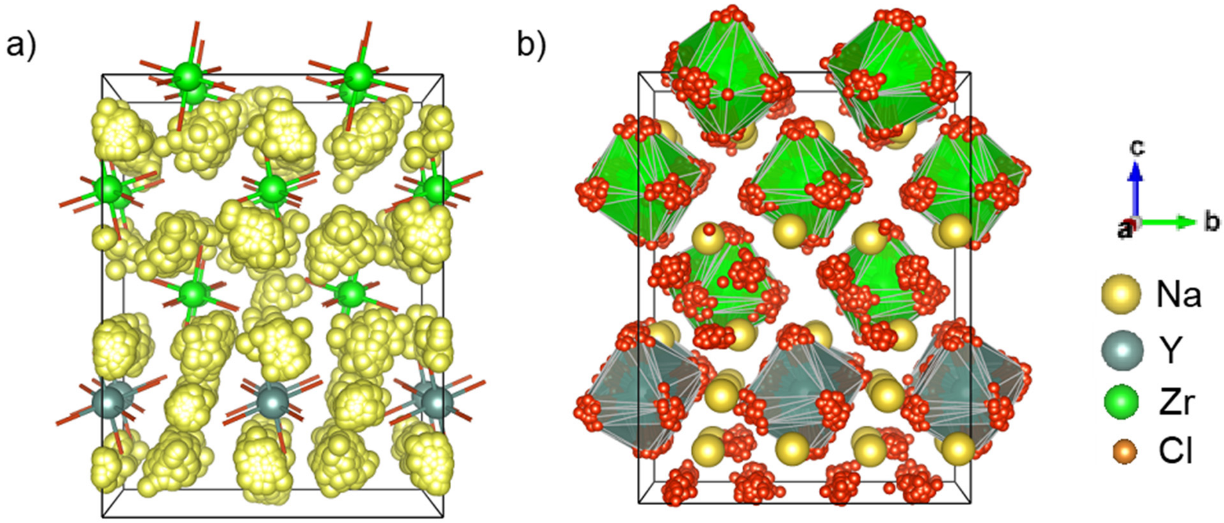


**Supplementary Figure 10 | $Na^+$ and $Cl^-$ trajectories at 600 K for volume-constrained $Na_{2.25}Y_{0.25}Zr_{0.75}Cl_6$. a,** Localized $Na^+$ hopping and **b**, Map of Cl trajectories over a 50 ps time interval for NYZC0.75 with the unit cell constrained to the volume of NYC; polyhedral rotation is more restricted.

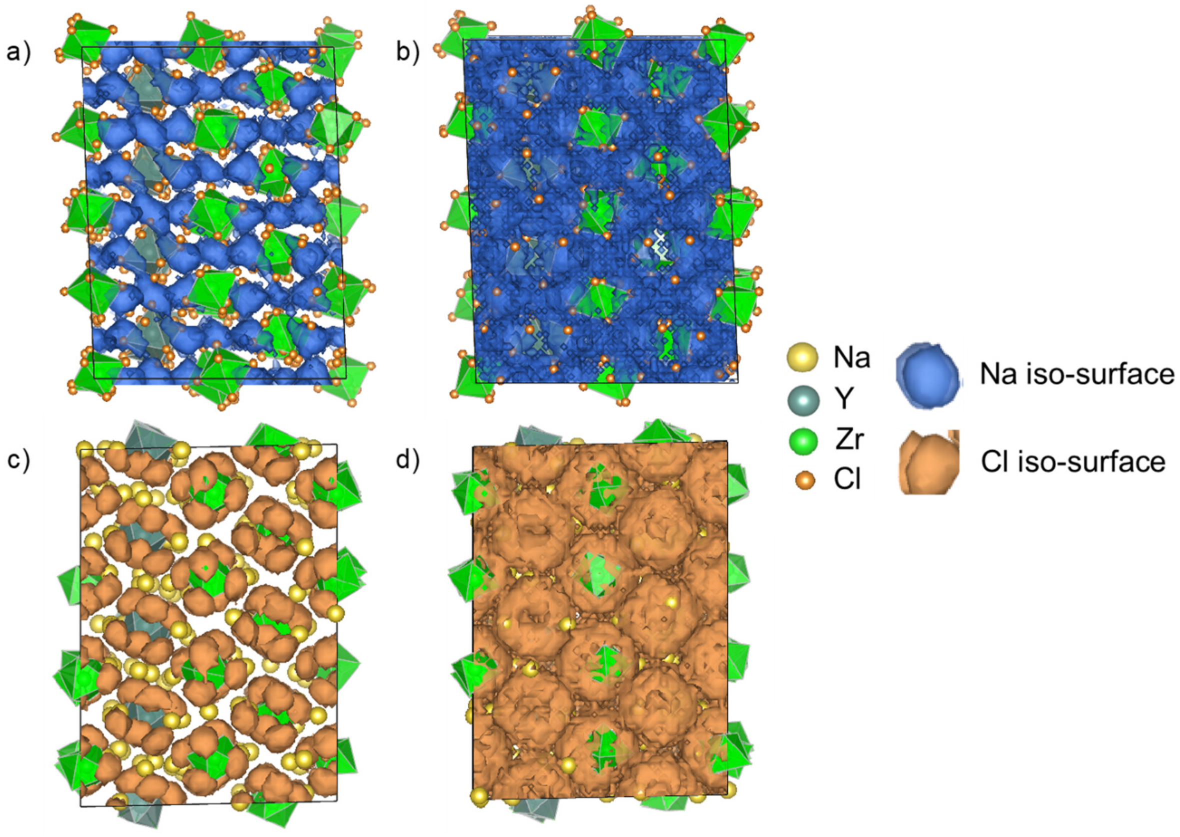


**Supplementary Figure 11 | $Na^+$ diffusion topology and octahedra rotation of $Cl^-$ below (500K) and above (550K) the transition point.** The trajectories are obtained from the ML-IAP MD simulations of $Na_{2.25}Y_{0.25}Z_{0.75}Cl_6$ for 10 ns. Plots of the probability density (isosurface value = 5 x $10^{-4}$) of $Na^+$ in $Na_{2.25}Y_{0.25}Z_{0.75}Cl_6$, **a,** T = 500 K **b**, T = 550 K; $Cl^-$ in $Na_{2.25}Y_{0.25}Z_{0.75}Cl_6$ at **c,** T = 500 K **d**, T = 550 K.

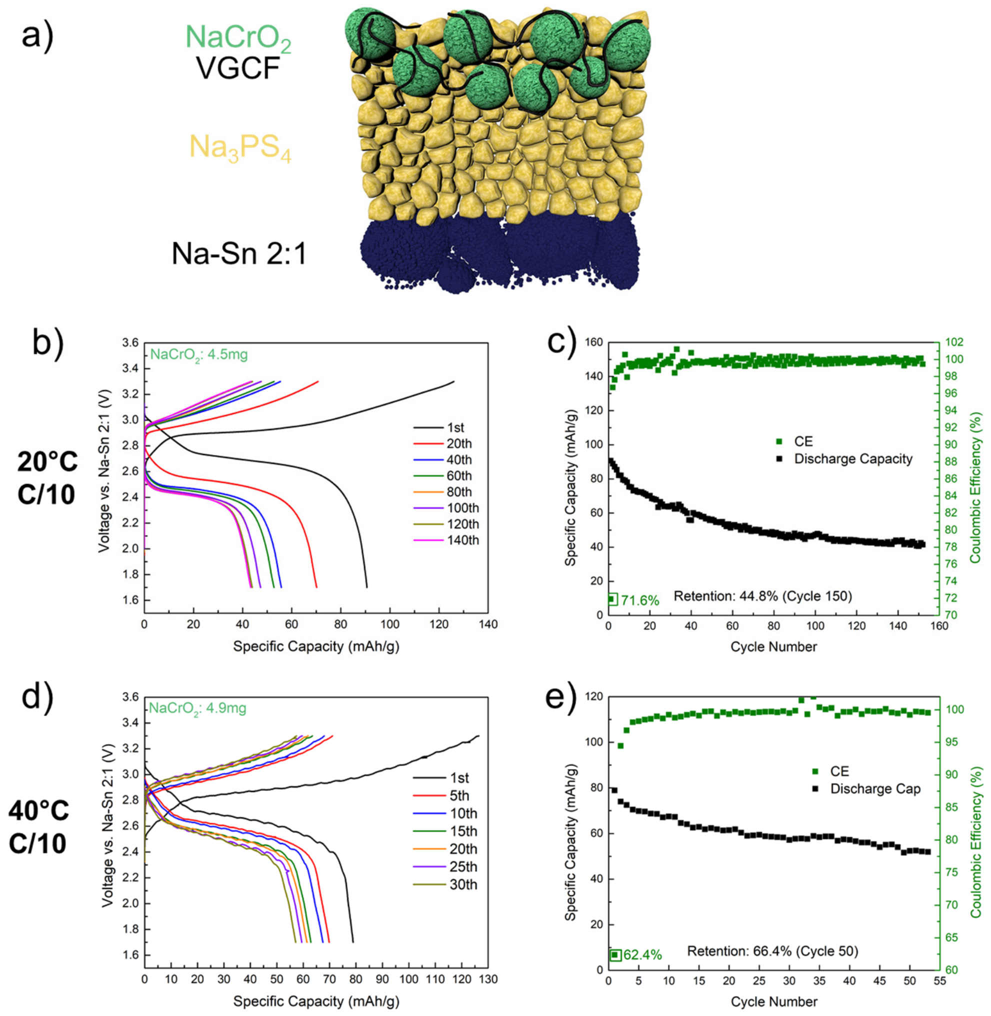


**Supplementary Figure 12 | Electrochemical performance of the NPS SSSBs. a**, Cell schematic. Voltage profile and specific capacity as a function of cycle number of this cell configuration, respectively, running at **b-c**, 20°C and C/10, and **d-e,** 40°C and C/10. Gradual capacity fade is observed as the capacity retention is 44.8% after 150 cycles (20°C) and 66.4% after 50 cycles (40°C), revealing the instability of NPS when paired with $NaCrO_2$.

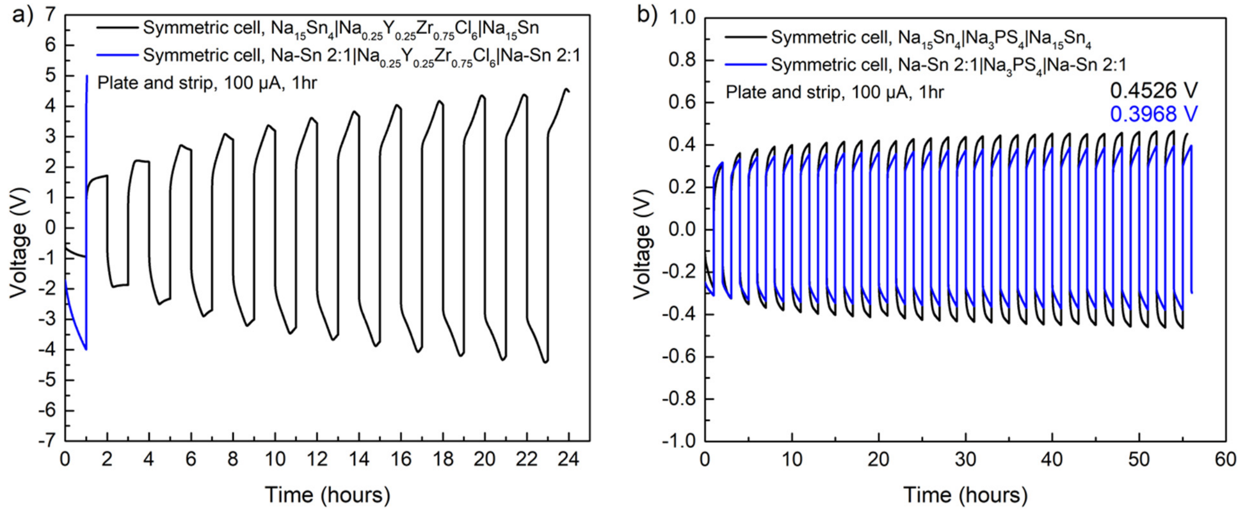


**Supplementary Figure 13 | Symmetric Cell Plating and Stripping. a**, Symmetric cell plating and stripping of $Na_{2.25}Y_{0.25}Zr_{0.75}Cl_6$ with $Na_{15}Sn_4$ and Na-Sn 2:1 electrodes. **b**, Symmetric cell plating and stripping of $Na_3PS_4$ with $Na_{15}Sn_4$ and Na-Sn 2:1 electrodes. High impedance or steady impedance growth is shown for $Na_{2.25}Y_{0.25}Zr_{0.75}Cl_6$ with Na-Sn anodes as opposed to $Na_3PS_4$; $Na_3PS_4$ is more stable when paired with Na-Sn, consistent with the EC window calculations.

**Supplementary Note 4**

To explore a reduction in overall cell impedance, a rate capability test was conducted on a NYZC0.75 cell at room temperature and one that used half the amount of NPS (37.5mg). Such a cell was fabricated on top of a stainless-steel current collector (600mg), that served as the cell support, as opposed to the NPS-supported configuration. The results, along with the corresponding Nyquist plots of the cells, are shown in Supplementary Figure 13. The reduction in the amount of NPS has a direct effect on the rate performance of the cell; at room temperature, the cell could even run at 1C. The Nyquist plots showed that the electrolyte contributions to cell impedance (designated as $R_b$ and $R_{int}$) were reduced by half (in total) for the stainless-steel supported cell, while the $R_{ct}$ component from the cathode remained about the same. This suggests that optimization of the ASSB form factor, notably with a thin electrolyte layer prepared by solution casting, would be a very promising avenue to explore for future work.[6]

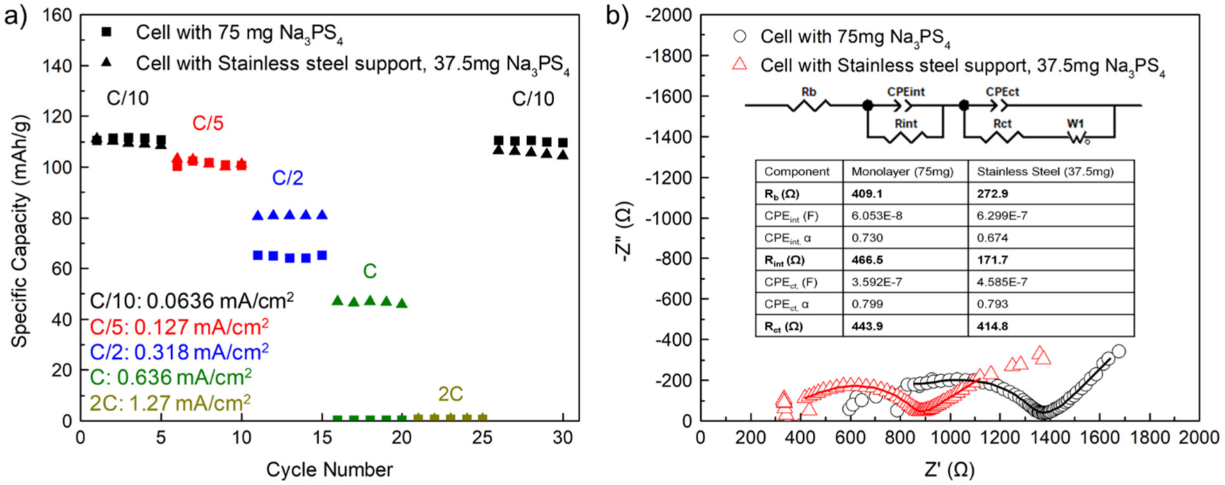


| Component | Monolayer (75mg) | Stainless Steel (37.5mg) |
|---|---|---|
| $R_b$ (Ω) | 409.1 | 272.9 |
| $CPE_{int}$ (F) | 6.053E-8 | 6.299E-7 |
| $CPE_{int}$ α | 0.730 | 0.674 |
| $R_{int}$ (Ω) | 466.5 | 171.7 |
| $CPE_{ct}$ (F) | 3.592E-7 | 4.585E-7 |
| $CPE_{ct}$ α | 0.799 | 0.793 |
| $R_{ct}$ (Ω) | 443.9 | 414.8 |

**Supplementary Figure 14 | Rate Capability. a**, Rate capability test (conducted at room temperature) of the NYZC0.75 cell and the stainless steel-supported NYZC0.75 cell and **b**, Nyquist plots and fit for the respective cells. Reducing the NPS amount by half was associated with a reduction of the overall cell impedance by half, improving the rate capability of the cell.

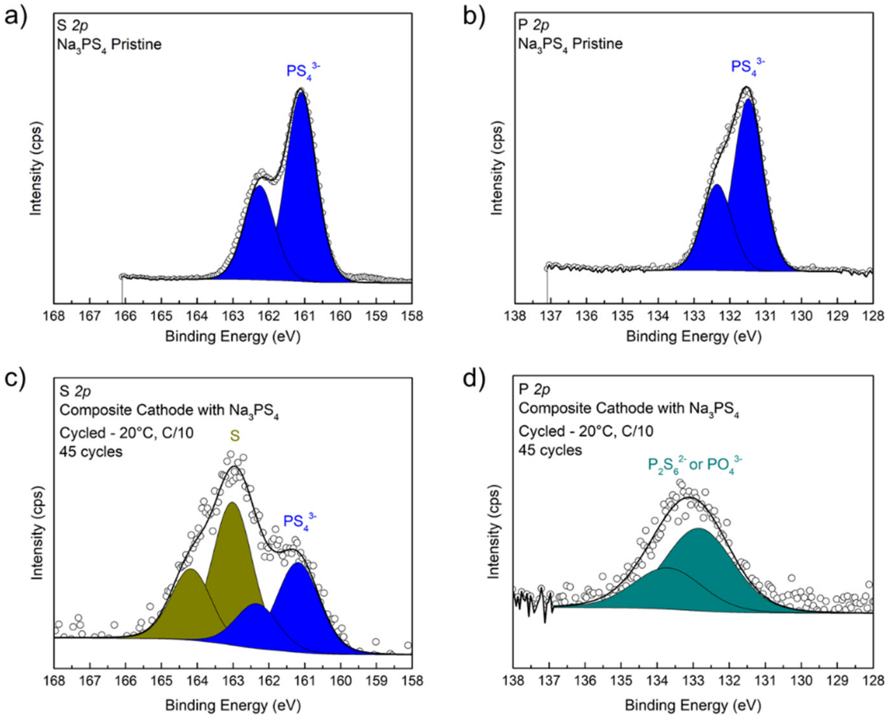


**Supplementary Figure 15 | XPS of the cycled $Na_3PS_4$-containing composite cathode. a**, S *2p* and **b**, P *2p* binding energy regions of pristine $Na_3PS_4$. **c**, S *2p* and **d**, P *2p* binding energy regions of the composite cathode of the cycled room temperature NPS-only cell. In the cycled samples, the presence of new peaks in the S *2p* region, corresponding to sulfur, and shifted peaks in the P *2p* region, indicate the electrochemical oxidation of $Na_3PS_4$ at the cathode interface.

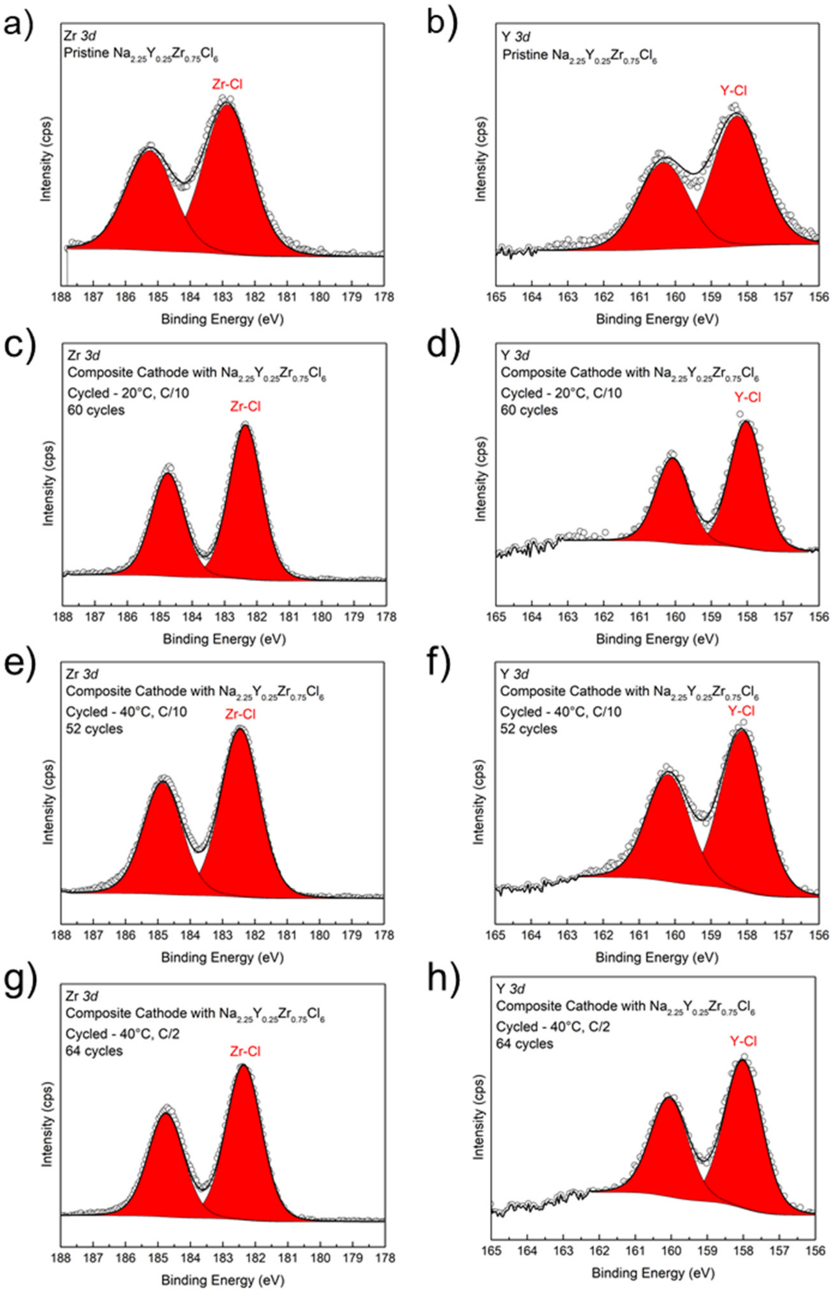


**Supplementary Figure 16 | XPS of the Cycled $Na_{2.25}Y_{0.25}Zr_{0.75}Cl_6$-containing Composite Cathode.** Zr *3d* and Y *3d* binding energies, respectively, for **a-b**, Pristine $Na_{2.25}Y_{0.25}Zr_{0.75}Cl_6$, **c-d**, Room temperature cycled $NaCrO_2$:$Na_{2.25}Y_{0.25}Zr_{0.75}Cl_6$:VGCF composite cathode, **e-f**, 40°C cycled $NaCrO_2$:$Na_{2.25}Y_{0.25}Zr_{0.75}Cl_6$:VGCF composite cathode, and **g-h**, 40°C cycled $NaCrO_2$: $Na_{2.25}Y_{0.25}Zr_{0.75}Cl_6$:VGCF composite cathode at a rate of C/2. No extra peaks or significant shifts in the Zr *3d* or Y *3d* peaks are observed so the Zr-Cl and Y-Cl bonds are maintained throughout cycling.

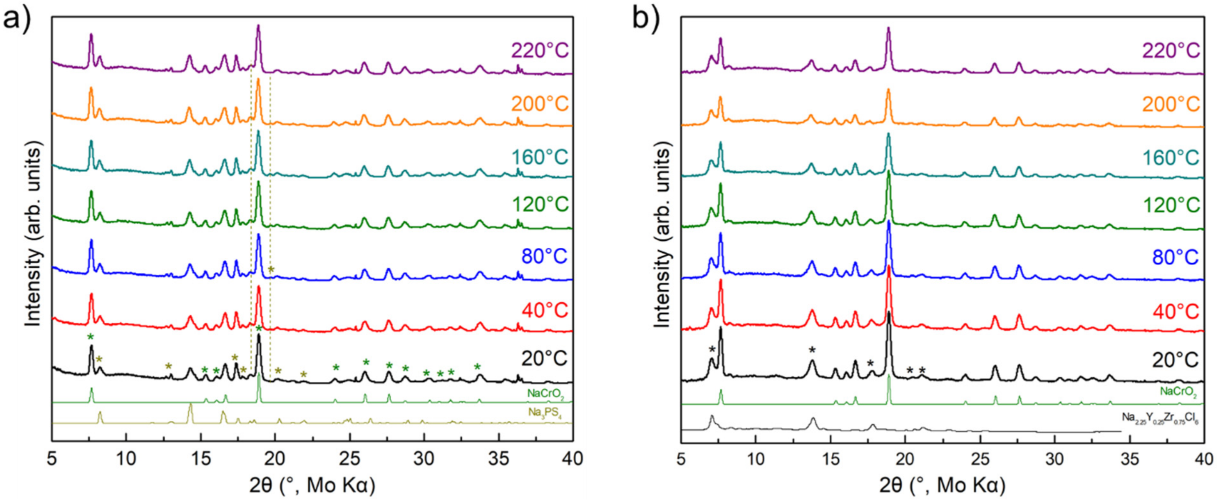


**Supplementary Figure 17 | Temperature-dependent XRD**. **a**, 1:1 NPS:$NaCrO_2$ mixture and **b**, 1:1 NYZC0.75:$NaCrO_2$ mixture. No extra peaks were seen in either case, consistent with the low reaction energy from computational results. The low initial CE and the capacity fade of the $Na_3PS_4$-only cell thus comes from the electrochemical oxidation of $Na_3PS_4$ at high voltages.

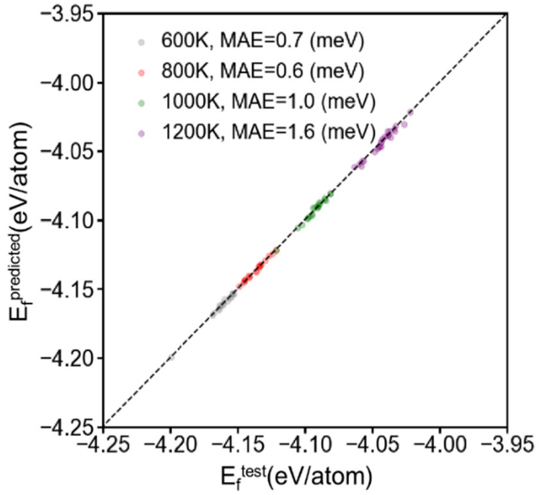


**Supplementary Figure 18 | Mean absolute error (MAE) Comparison.** MAE of the energies and forces for moment tensor potential (MTP) versus DFT for $Na_{2.25}Y_{0.25}Zr_{0.75}Cl_6$.

**Supplementary Table 5 | Mean absolute error (MAE).** MAE of the energies and forces between the training and test data sets.

| | $MAE_{energies}$ (meV/atom) | $MAE_{forces}$ (meV/Å) |
|---|---|---|
| Training | 0.855 | 61.4 |
| Test | 0.966 | 63.5 |

**Supplementary Information References**


1. Liao, W. & Dronskowski, R. Trisodium yttrium(III) hexachloride. *Acta Crystallogr. Sect. E Struct. Rep. Online* **60**, i72–i73 (2004).
2. Asano, T. *et al.* Solid Halide Electrolytes with High Lithium-Ion Conductivity for Application in 4 V Class Bulk-Type All-Solid-State Batteries. *Adv. Mater.* **30**, 1803075 (2018).
3. Schäfer, G. F. The crystal structures of $Na_2TiF_6$ and $Na_2SiF_6$. *Z. Für Krist. - Cryst. Mater.* **175**, 269–276 (2015).
4. Schlem, R., Banik, A., Eckardt, M., Zobel, M. & Zeier, W. G. $Na_{3-x}Er_{1-x}Zr_xCl_6$ - A halide-based fast sodium-ion conductor with vacancy-driven ionic transport. *ACS Appl. Energy Mater.* (2020) doi:10.1021/acsaem.0c01870.
5. Sleigh, C., Pijpers, A. P., Jaspers, A., Coussens, B. & Meier, R. J. On the determination of atomic charge via ESCA including application to organometallics. *J. Electron Spectrosc. Relat. Phenom.* **77**, 41–57 (1996).
6. Tan, D. H. S. *et al.* Enabling Thin and Flexible Solid-State Composite Electrolytes by the Scalable Solution Process. *ACS Appl. Energy Mater.* **2**, 6542–6550 (2019).
